# Zinc-finger protein 471 suppresses gastric cancer through transcriptionally repressing downstream oncogenic PLS3 and TFAP2A

**DOI:** 10.1038/s41388-018-0220-5

**Published:** 2018-04-03

**Authors:** Lei Cao, Shiyan Wang, Yanquan Zhang, Ka-Chun Wong, Geicho Nakatsu, Xiaohong Wang, Sunny Wong, Jiafu Ji, Jun Yu

**Affiliations:** 1 Institute of Digestive Disease and Department of Medicine and Therapeutics, State Key laboratory of Digestive Disease, Li Ka Shing Institute of Health Sciences, The Chinese University of Hong Kong, Hong Kong, China; 2 Department of Computer Science, City University of Hong Kong-Kowloon Tong, Hong Kong, China; 3 Key laboratory of Carcinogenesis and Translational Research (Ministry of Education), Department of Gastrointestinal Surgery, Beijing Cancer Hospital and Institute, Beijing, China

**Keywords:** DNA methylation, Gastric cancer, Transcription

## Abstract

Zinc-finger protein 471 (ZNF471) was preferentially methylated in gastric cancer using promoter methylation array. The role of ZNF471 in human cancer is unclear. Here we elucidated the functional significance, molecular mechanisms and clinical impact of ZNF471 in gastric cancer. ZNF471 mRNA was silenced in 15 out of 16 gastric cancer cell lines due to promoter hypermethylation. Significantly higher ZNF471 promoter methylation was also observed in primary gastric cancers compared to their adjacent normal tissues (*P* *<* 0.001). ZNF471 promoter CpG-site hypermethylation correlated with poor survival of gastric cancer patients (*n* = 120, *P* *=* 0.001). Ectopic expression of ZNF471 in gastric cancer cell lines (AGS, BGC823, and MKN74) significantly suppressed cell proliferation, migration, and invasion, while it induced apoptosis in vitro and inhibited xenograft tumorigenesis in nude mice. Transcription factor AP-2 Alpha (TFAP2A) and plastin3 (PLS3) were two crucial downstream targets of ZNF471 demonstrated by bioinformatics modeling and ChIP-PCR assays. ZNF471 directly bound to the promoter of TFAP2A and PLS3 and transcriptionally inhibited their expression. TFAP2A and PLS3 showed oncogenic functions in gastric cancer cell lines. Moreover, ZNF471 recruited KAP1 to the promoter of the target genes, thereby inducing H3K9me3 enrichment for transcriptional repression and inhibition of oncogenic TFAP2A and PLS3. In conclusion, ZNF471 acts as a tumor suppressor in gastric cancer by transcriptionally inhibiting downstream targets TFAP2A and PLS3. KAP1 is a co-repressor of ZNF471 at the promoter of the target genes. The promoter CpG-site methylation is an independent prognostic factor for overall survival of gastric cancer patients.

## Introduction

Gastric cancer remains the fifth most common malignancy all over the world, after cancers of lung, breast, colorectum, and prostate [1]. From the aspect of geographic distribution, Eastern Asia demonstrates the highest estimated rates for both incidence and mortality. Development of gastric cancer relies on a gradual accumulation of multiple genetic and epigenetic alterations, most of which are yet to be explored. In gastric cancer, suppressors like CDH1, MLH1, and CDKN2A are commonly silenced due to the promoter methylation [2–6].

Zinc-finger proteins (ZFPs), which compose the largest family of transcription factors, tend to be silenced by promoter hypermethylation in gastric cancer [5]. Aberrant inactivation of ZFPs has been reported to contribute to irregular gene expression and tumor development [7]. In combination of our in-house gastric cancer DNA methylation (450k) data (gastric cancer cell lines AGS, MGC803, MKN45, normal gastric tissue, and normal gastric epithelial GES1; data unpublished) and TCGA gastric cancer DNA methylation (450k) data, we found that ZNF471 is one the most significantly hypermethylated zinc-finger protein genes, with low mRNA expression in gastric cancer as compared with normal gastric tissue or tumor adjacent tissues (*q* value = 0.0003). Frequent methylation of ZNF471 in cancer has also been supported by published epigenetic analysis on colorectal and squamous cell carcinoma [8, 9]. ZNF471 is one of the KRAB C2H2-type ZFP family, containing a KRAB-domain at N terminal and 15 zinc fingers at C-terminal. KRAB-ZFP family members have been reported to induce transcriptional silencing by binding to the promoter of target genes through a C2H2 zinc-finger domain, and recruiting co-repressor to KRAB-domain [10, 11]. Investigations of the functional significance of KRAB-ZFP family members in gastric cancer may lead to a better understanding of the molecular mechanisms of gastric carcinogenesis as well as identify potential targets for the diagnosis and treatment of gastric cancer. As a member of KRAB-ZFP family, the role of ZNF471 in human gastric cancer is unclear. Hence, in this study, we elucidated the expression profile, epigenetic regulation, biological function, downstream effectors, promoter co-regulator, and clinical impact of ZNF471 in gastric cancer.

## Results

### ZNF471 is downregulated or silenced in gastric cancer by promoter methylation

**Fig. 1:**
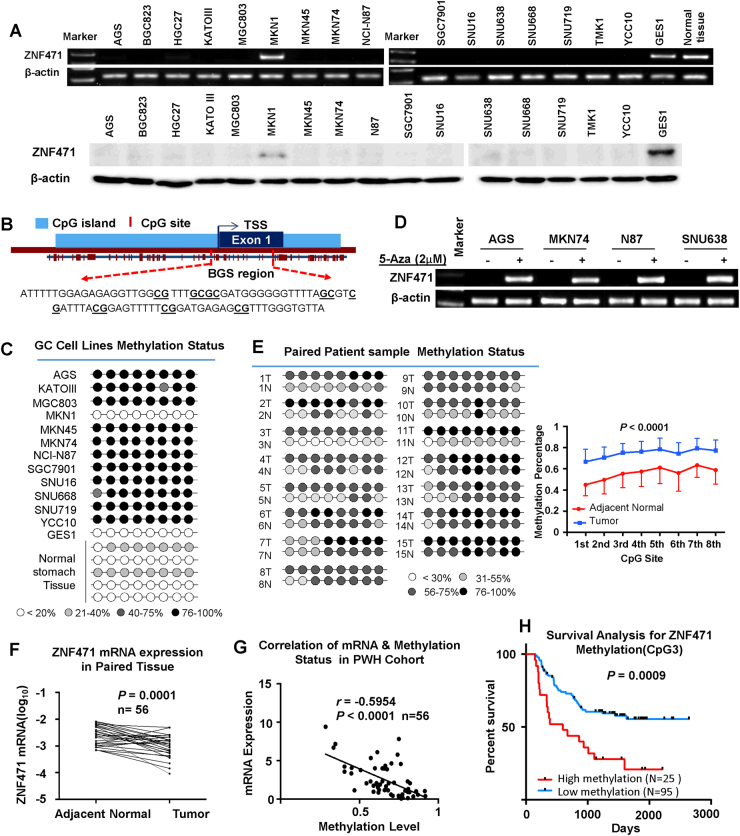
The silence or downregulation of ZNF471 in gastric cancer was governed by promoter methylation and predicted poor survival. **a** Upper, mRNA expression of ZNF471 in gastric cancer cell lines by PCR; lower, protein expression of ZNF471 in gastric cancer cell lines by Western blot. **b** The localization of CpG sites for bisulfide genome sequencing (BGS). **c** The methylation status of the randomly selected CpG sites of ZNF471 promoter in gastric cancer cell lines. **d** ZNF471 mRNA expression level after 5-Aza treatment in gastric cancer cell lines. **e** The methylation status of ZNF471 promoter in paired patient samples (*n* = 15). **f** The mRNA expression of ZNF471 in paired patient samples (*n* = 56). **g** The correlation between ZNF471 mRNA level and its promoter methylation status (*n* = 56). **h** Kaplan–Meier curves of patients with gastric cancer, stratified by CpG-site 3 in ZNF471 promoter methylation status. Data are expressed as mean ± S.D.

Both mRNA and protein expression of ZNF471 were silenced or downregulated in 15 out of 16 gastric cancer cell lines, while it was detected in MKN1, GES1 (a normal gastric epithelia cell line) as well as normal gastric tissue (Fig. 1a). Bisulfite genomic sequencing (BGS) results indicate that all the gastric cancer cell lines but MKN1 demonstrated hypermethylation at CpG sites in ZNF471 promoter region, whereas very low methylation was found in GES1 and normal gastric samples (Fig. 1b, c), consistent with ZNF471 expression in cell lines and gastric tissues. To further confirm the results, randomly selected cell lines with silenced ZNF471 level (AGS, MKN74, N87, and SNU638) were treated with 5-Aza (a methyltransferase inhibitor) with subsequent ZNF471 mRNA level examination. Restored mRNA expression of ZNF471 was observed (Fig. 1d), indicating that DNA hypermethylation at ZNF471 promoter region is involved in its transcriptional silencing in gastric cancer.

### ZNF471 promoter methylation is associated with poor survival for patients with gastric cancer

To estimate the clinical importance of promoter methylation level of ZNF471 in gastric cancer, we quantified the DNA methylation level of CpG sites in ZNF471 promoter in paired gastric tumors and tumor adjacent tissues by BGS. Results demonstrated that DNA methylation of ZNF471 promoter was significantly higher in gastric cancer [12] compared with paired adjacent tissues (Fig. 1e). In addition, the mRNA level of ZNF471 was significantly decreased in gastric tumor tissues compared with tumor adjacent tissues (*n* = 56, *P* *=* 0.0001) (Fig. 1f, Figure S1A). Higher protein expression of ZNF471 was detected in 6 out of 10 paired gastric tumors than adjacent tissues (*P* = 0.05, Figure S1A). A negative association was displayed between ZNF471 promoter methylation and the matched mRNA expression (*n* = 56, *r* *=* −0.5945, *P* *<* 0.0001) (Fig. 1g), which was further validated in TCGA data (Pearson’s *r* *=* −0.64, *P* *=* 8.6e−40) (Figure S1B).

With 120 gastric cancer patients (Supplementary Table S1), we evaluated the potential clinical impact of ZNF471 promoter methylation using a region harboring eight CpG sites. Univariate Cox regression analysis revealed that the median value of 8-CpG-site methylation was associated with overall survival for gastric cancer patients, high methylation positively correlates with poor survival (hazard ratios (HR) 1.27; 95% confidence interval (CI): 0.968–1.7; *P* *=* 0.084) (Table 1). Interestingly, the methylation status of five individual CpG sites of ZN471 promoter, i.e., CpG1 (*P* *=* 0.042), CpG2 (*P* *=* 0.044), CpG3 (*P* *=* 0.001), CpG4 (*P* *=* 0.038), and CpG7 (*P* = 0.041) also had significant association with overall survival (Table 1). In addition, gender (*P* *=* 0.035) and TNM stage (*P* *<* 0.001) were found to have statistically significant associations with overall survival (Table 1). After adjustment of gender and TNM stage, hypermethylation of ZNF471 promoter (median value) was associated with poorer survival of gastric cancer patients (HR 2.315; 95% CI: 1.307–4.099; *P* *=* 0.004) (Table 1). Multivariate cox regression analysis also demonstrated the prognostic value of single CpG-site CpG1, CpG3, CpG4, and CpG7 (Table 1). Among all, CpG3 of ZNF471 promoter had the predominant prognostic role for gastric cancer patients with the most significant *P* value (HR 2.616; 95% CI: 1.491–4.590; *P* *=* 0.001) (Table 1). Comparison of clinicopathological features between patients with high and low ZNF471 methylation revealed that high methylation level of ZNF471 positively correlated with gastric cancer metastasis (*P* = 0.0039) (Supplementary Table S2).

**Table 1:** Univariate and multivariate Cox analyses of potential prognostic factors for patients with gastric cancer

Viable	Univariate Cox regression analysis		Multivariate Cox regression analysis	
	HR (95% CI)	*p* value	HR (95% CI)	*p* value
Age	0.866 (0.509 to 1.473)	0.39		
<=65	1.264 (0.743 to 2.15)			
<65	1.00			
Gender				
Female (*n* = 39)	1.763 (1.040 to 2.987)	0.035	2.259 (1.317 to 3.873)	0.003
Male (*n* = 81)	1.00		1.00	
TNM stage				
I/II (*n* = 35)	0.232 (0.105 to 0.512)	0.000	0.236 (0.106 to 0.524)	0.000
III/IV (*n* = 85)	1.00		1.00	
Differentiation				
Low	0.925 (0.468 to 1.830)	0.823		
Medium/High	1.00			
Median value of 8-CpG sites				
High (*n* = 26)	1.908 (1.083 to 3.362)	0.025	2.315 (1.307 to 4.099)	0.004
Low (*n* = 94)	1.00		1.00	
CpG-site 1				
High (*n* = 26)	1.342 (1.011 to 1.8)	0.042	1.826 (1.025 to 3.252)	0.041
Low (*n* = 94)	1.00		1.00	
CpG-site 2				
High (*n* = 15)	1.402 (1.010 to 1.948)	0.044	Failed	
Low (*n* = 105)	1.00			
CpG-site 3				
High (*n* = 25)	1.595 (1.212 to 2.100)	0.001	2.616 (1.491 to 4.590)	0.001
Low (*n* = 95)	1.00		1.00	
CpG-site 4				
High (*n* = 26)	1.349 (1.016 to 1.79)	0.038	1.898 (1.069 to 3.370)	0.029
Low (*n* = 94)	1.00		1.00	
CpG-site 5				
High (*n* = 17)	1.207 (0.913 to 1.6)	0.186		
Low (*n* = 103)	1.00			
CpG-site 6				
High (*n* = 29)	1.148 (0.9 to 1.532)	0.348		
Low (*n* = 91)	1.00			
CpG-site 7				
High (*n* = 44)	1.309 (1.010 to 1.696)	0.041	2.076 (1.220 to 3.503)	0.007
Low (*n* = 76)	1.00		1.00	
CpG-site 8				
High (*n* = 45)	1.226 (0.46 to 1.589)	0.124		
Low (*n* = 75)	1.00			

The Kaplan–Meier method was used to produce survival curves stratified by ZNF471 methylation status. High methylation level of CpG3 and 8-CpG-site median or other single CpG-site (CpG1, CpG4, and CpG7) predicted shorter survival time for gastric cancer patients (Fig. 1h, Figure S1C). After stratification by TNM stage, gastric cancer patients with high CpG3 methylation in ZNF471 promoter demonstrated significantly shorter survival in stage III/IV (*P* = 0.003), but not in stage I/II (Figure S1D). The prognostic value of ZNF471 methylation has also been supported by survival analysis of TCGA data (Figure S2). Taken together, hypermethylation of ZNF471 functioned as an independent marker for poor gastric cancer prognosis.

### ZNF471 suppresses gastric cancer via inhibiting cell proliferation and inducing apoptosis and cell cycle arrest

**Fig. 2:**
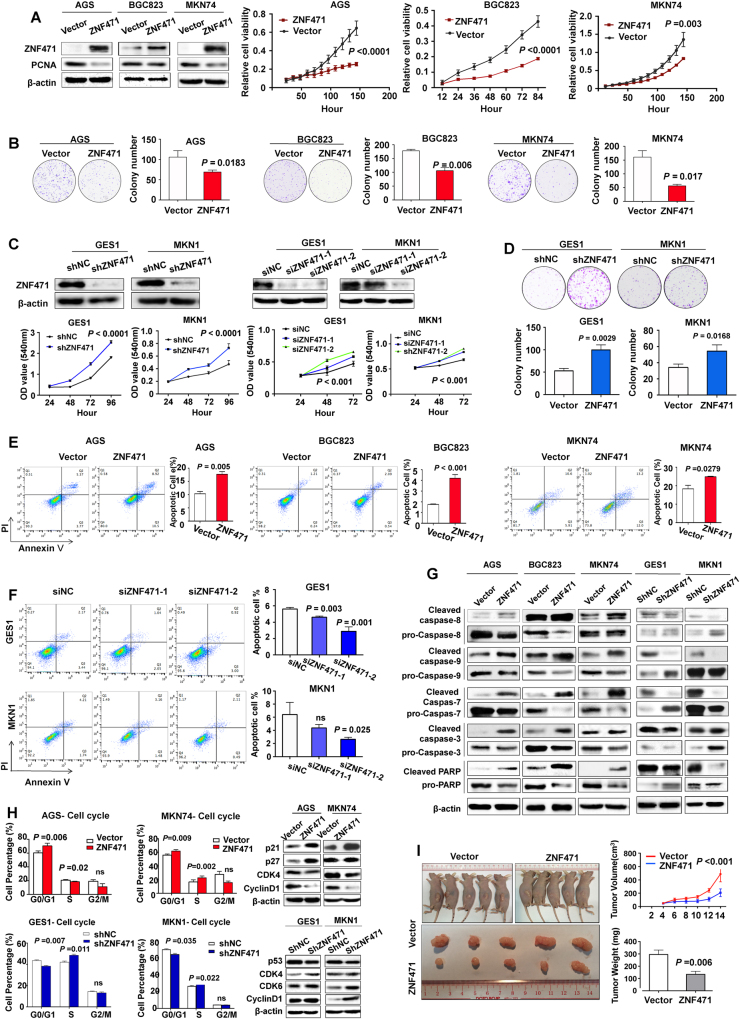
Ectopic expression of ZNF471 triggered cell apoptosis and cell proliferation inhibition. **a** Left, ZNF471 and PCNA expression by Western blot after ZNF471 overexpression; right, the effect of ZNF471 overexpression on cell viability determined by MTT assay in AGS, BGC823, and MKN74. **b** The effect of ZNF471 overexpression on cell colony formation ability in AGS, BGC823, and MKN74 cells. **c** ZNF471 knockdown efficiency and effect on cell viability in GES1 and MKN1 by shRNA (left) or siRNA (right). **d** The effect of ZNF471 knockdown on cell colony formation ability in GES1 and MKN1 cells. **e** The effect of ZNF471 overexpression on cell apoptosis measured with Annexin V-PE and 7-aminoactinomycin (7-AAD) double staining by flow cytometer in AGS, BGC823, and MKN74. **f** The effect of ZNF471 knockdown on cell apoptosis in GES1 and MKN1. **g** The protein level of cell apoptosis markers upon ZNF471 overexpression or knockdown by Western blot. **h** Upper, the effect of ZNF471 overexpression on cell cycle distribution and cell cycle regulators by Western blot; lower, the effect of ZNF471 knockdown on cell cycle distribution and cell cycle regulators by Western blot. **i** The effect of ZNF471 on tumor formation ability in nude mice xenograft model. Data are expressed as mean ± S.D.

To investigate the functional role of ZNF471 in gastric cancer, we overexpressed ZNF471 in AGS, BGC823, and MKN74. Overexpression of ZNF471 resulted in significantly reduced proliferating cell nuclear antigen (PCNA) protein expression, inhibited cell growth (Fig. 2a) as well as reduced colony formation (Fig. 2b). In contrast, knockdown of endogenous ZNF471 in GES1 and MKN1 cells by siRNA or shRNA increased cell viability (Fig. 2c) and colony formation abilities (Fig. 2d). Consistent with cell viability assay, overexpression of ZNF471 prolonged cell doubling time, while knockdown of ZNF471 shortened cell doubling time (Figure S3).

In addition to repressing cell proliferation, ZNF471-triggered gastric cancer cell apoptosis, with a significant increase in total apoptosis proportion (about 6% in AGS, 3% in BGC823, and 8% in MKN74, respectively) (Fig. 2e). On the other hand, apoptotic cell percentage was decreased with ZNF471 knockdown in GES1 and MKN1 (Fig. 2f). Cell apoptosis was activated through caspase cascade, as the enhanced level of cleaved caspase-9, capase-8, caspase-3, caspase-7, and poly ADP-ribose polymerase (PARP) was observed (Fig. 2g). Overexpressing ZNF471-induced G0/G1 phase cell cycle arrest, which was confirmed by decreased key G0/G1 cell cycle regulators, cyclin D1 and CDK4 (Fig. 2h). Knockdown of ZNF471 promoted G1/S cell cycle transition, as confirmed by enhanced protein expression of CDK6, CKD4, and cyclin D1 (Fig. 2h). Moreover, in the subcutaneous xenograft mice model, stable overexpression of ZNF471 in BGC823 cells suppressed tumor growth and volume (Fig. 2i).

### ZNF471 attenuated the gastric cancer cell migration and invasion

**Fig. 3:**
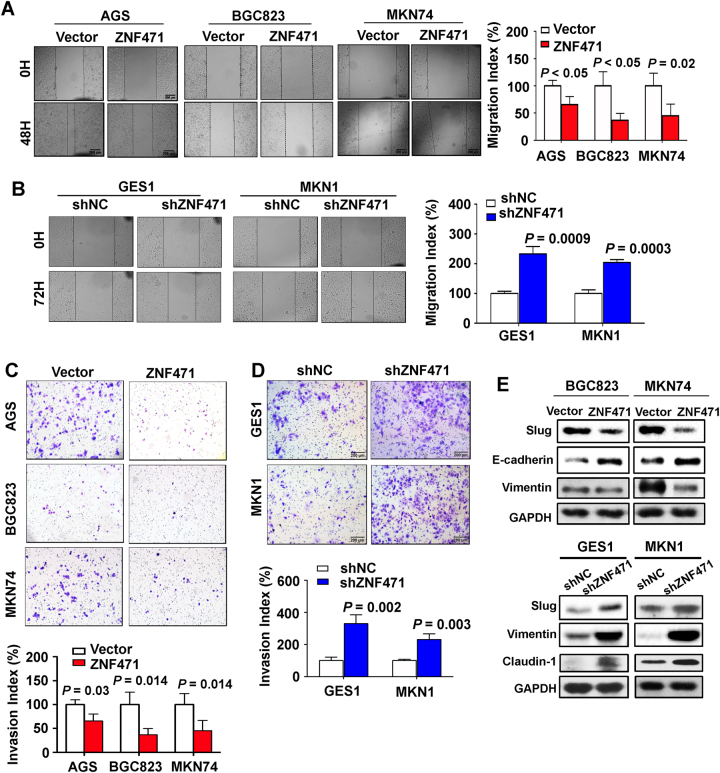
ZNF471 attenuated the gastric cancer cell migration and invasion. **a** Wound healing assay for the evaluation of ZNF471 overexpression on AGS, BGC823, and MKN74 migration ability. **b** Wound healing assay for the evaluation of ZNF471 knockdown on GES1 and MKN1 migration ability. **c** The cell invasion ability measured by Matrigel-coated trans-well upon ZNF471 overexpression in AGS, BGC823, and MKN74. **d** The cell invasion ability measured by Matrigel-coated trans-well upon ZNF471 knockdown with shRNA in GES1 and MKN1. **e** The effect of ZNF471 expression on protein level of key epithelial–mesenchymal transition (EMT) markers by Western blot. Data are expressed as mean ± S.D.

To understand the functional role of ZNF471 in gastric cancer migration and invasion, we performed wound healing and Matrigel invasion assay. Quantitative analysis of wound healing assay revealed that the wound closure speed was slower in ZNF471-overexpressing cells than in control cells (Fig. 3a). Although, the wound closure speed in GES1 and MKN1 were accelerated after ZNF471 knockdown (*P* < 0.05) (Fig. 3b). Matrigel invasion assay demonstrated that re-expression of ZNF471 markedly attenuated cell invasion in AGS, BGC823, and MKN74 cell lines (*P* < 0.05) (Fig. 3c). On the other hand, knockdown of ZNF471 increased cell invasion ability in GES1 and MKN1 cell lines (*P* < 0.05) (Fig. 3d). Western blot demonstrated the reduced expression of Vimentin and Slug as well as increased E-cadherin, key epithelial–mesenchymal transition (EMT) markers, when re-expressing ZNF471. Meanwhile, increased level of Vimentin, Slug, and Claudin-1 was observed in GES1 and MKN1 with ZNF471 knockdown (Fig. 3e). These findings indicate that ZNF471 suppresses the migration and invasion of gastric cancer cells, acting as an indirect inhibitor of the key EMT regulators.

### Genome-wide identification of transcriptional targets for ZNF471 by bioinformatics modeling

**Fig. 4:**
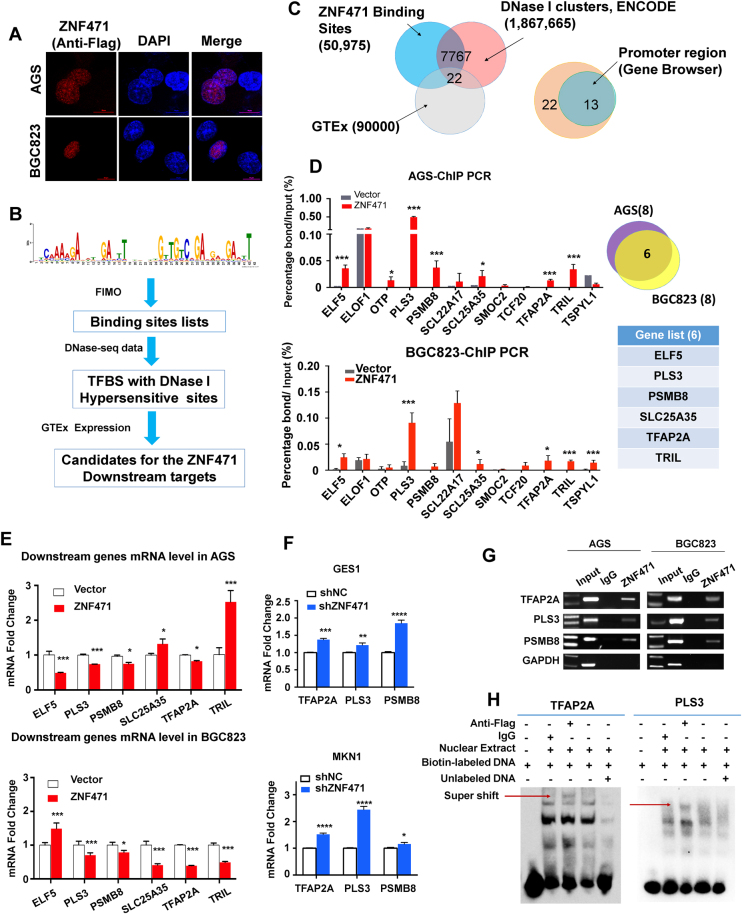
Identification of direct transcriptional targets for ZNF471 with application of bioinformatics modeling. **a** Confocal image of ZNF471 cellular localization by immunofluorescence. **b** Workflow of ZNF471 transcriptional targets identification. **c** The candidate genes selected with whole genome ZNF471 binding sites and gene transcription regulation database using Venn diagram. **d** ChIP-qPCR validation of candidate genes in AGS and BGC823 with re-expression of ZNF471. **e** The mRNA expression level of candidate genes upon ZNF471 overexpression in AGS and BGC823 by qPCR. **f** The mRNA expression level of TFAP2A, PLS3 and PSMB8 and upon ZNF471 knockdown in GES1 and MKN1 by qPCR. **g** RT-PCR validation of TFAP2A, PLS3, and PSMB8 enriched by ZNF471-ChIP at promoter region. **h** EMSA performed with the biotin-labeled TFAP2A (left panel) or PLS3 (right panel) probes and 293T nuclear extracts with overexpressed ZNF47-Falg. Super shifts by anti-Flag antibody are indicated with arrows. Data are expressed as mean ± S.D. *P < 0.05, **P < 0.01, ***P < 0.001, ****P < 0.0001

Like other C2H2-ZFP members, ZNF471 may function as a transcription factor. Immunofluorescence staining showed that ZNF471 localized in the nucleus of gastric cancer cell lines (AGS and BGC823) with ectopic expression of ZNF471, supporting its potential role as a transcriptional regulator (Fig. 4a). We searched for the direct downstream binding targets of ZNF471 by predicting the binding motif with zinc-finger DNA motif recognition model [13] (Fig. 4b), which generated 50,975 candidate ZNF471 binding sites on the whole human genome (hg19). To filter out the true binding targets of ZNF471, we assessed the overlap of the ZNF471 binding sites with the published DNase I cluster data (1,867,665 reads generated from 125 cell lines of human tissues, Supplementary Table S3) for open chromatin status from ENCODE and the gene expression data from Genotype-Tissue Expression (GTEx) track, which narrowed the candidates down to 22 genes (Fig. 4c, Supplementary Table S4). Focusing on the transcription fact or function of ZNF471, we restricted the gene candidates to those with predicted binding sequences at ±2000 bp from the reference sequence (RefSeq) transcription start sites [14], further trimming down the number of candidates to thirteen (Fig. 4c). To validate the actual binding of ZNF471 at the promoter region of these predicted candidates, we performed ChIP-qPCR in AGS and BGC823 cell lines. We confirmed the direct binding of 8 candidates in AGS and BGC823 by ChIP-qPCR, respectively, with 6 genes consistently enriched by ZNF471 in both AGS and BGC823 (Fig. 4d), including TFAP2A, PLS3, E74 Like ETS Transcription Factor 5 (ELF5), Proteasome Subunit Beta 8(PSMB8), Solute Carrier Family 25 Member 35(SLC25A35), and TLR4 Interactor With Leucine Rich Repeats (TRIL) (Fig. 4d). To know the critical genes mediated by ZNF471 at transcriptional level, we detected the mRNA expression of 6 candidates by qPCR. TFAP2A, PLS3, and PSMB8 were consistently decreased in AGS and BGC823 cell lines upon overexpression of ZNF471 (Fig. 4e), but were increased under ZNF471 knockdown in GES1 and MKN1 (Fig. 4f). The binding of ZNF471 at the promoter region of TFAP2A, PLS3, and PSMB8 were confirmed by ChIP-PCR assay (Fig. 4g). To validate the binding activity and specificity of ZNF471 on downstream genes, we performed electrophoretic mobility shift assay (EMSA) using the promoter region of target genes as the probe. EMSA result demonstrated that the nuclear extracts containing ZNF471 protein induced bands shift for TFAP2A and PLS3 but not PSMB8 (Fig. 4h, Figure S4A). Further, the ZNF471 binding specificity was determined by supershift bands with anti-Flag antibody (Fig. 4h). The specificity of target gene promoters was supported by the competitive binding of unlabeled DNA probe to ZNF471, which prevented the binding of biotin-labeled probes (Fig. 4h). The binding affinity of ZNF471 was expressed as the dissociation constant *K*_D_ by quantification of interaction between ZNF471-expressing nuclear extracts and DNA probes. ZNF471 displayed similar biding affinity to TFAP2A (*K*_D_ = 388 ± 37.3 ng/µl) and PLS3 (*K*_D_ = 392 ± 28.78 ng/µl) (Figure S5).

### ZNF471 inhibited gastric cancer proliferation and metastasis by directly inhibiting TFAP2A and PLS3

**Fig. 5:**
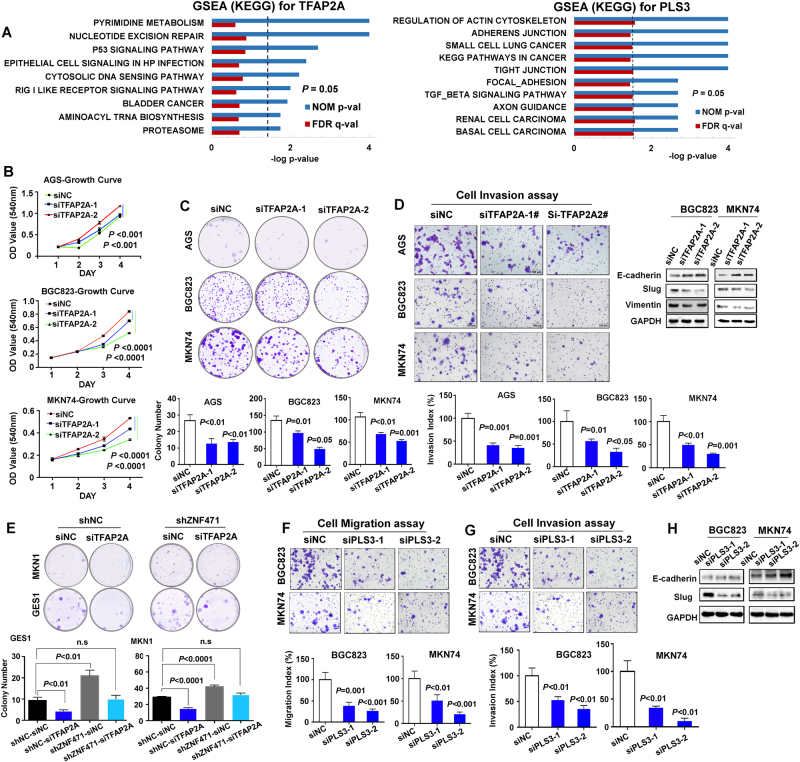
TFAP2A and PLS3, transcriptionally repressed by ZNF471, functioned as oncogenic factors. **a** The top 10 enriched pathways positively associated with high TFAP2A and PLS3 expression by GSEA using in TCGA Stomach Cancer gene expression RNAseq. **b** The effect of TFAP2A on cell viability in AGS, BGC823, and MKN74 upon knockdown by siRNA. **c** The effect of TFAP2A knockdown on cell colony formation ability in AGS, BGC823, and MKN74 cells. **d** Left, the effect of TFAP2A knockdown on cell invasion ability by Matrigel-coated trans-well assay in AGS, BGC823, and MKN74 cells; right, the expression of key EMT markers by Western blot. **e** Evaluation of dependency on TFAP2A by colony formation assay. The TFAP2A was further knocked-down by siRNA in ZNF471 knockdown cells. **f** The effect of PLS3 knockdown on cell migration ability by trans-well assay in BGC823 and MKN74 cells. **g** The effect of PLS3 knockdown on cell invasion ability by Matrigel-coated trans-well assay in BGC823 and MKN74 cells. **h** The expression of key EMT markers in BGC823 and MKN74 with PLS3 knockdown by Western blot. Data are expressed as mean ± S.D.

We did Gene Set Enrichment Analysis (GSEA) [15, 16] using TCGA Stomach Cancer gene expression RNAseq data and found that high expression level of either TFAP2A or PLS3 are associated with the activation of cancer-related pathway in gastric cancer patients (Fig. 5a); whereas the top positively enriched KEGG pathways for PSMB8 were less associated with cancer (Figure S4B). Taken the EMSA and GSEA results together, we therefore focused on the functional role of TFAP2A and PLS3 in gastric cancer. Knockdown of TFAP2A with two siRNA (Figure S4C) in AGS, BGC823, and MKN74 decreased cell proliferation (Fig. 5b), colony formation (Fig. 5c), and cell invasion ability (Fig. 5d). In keeping with this, Western blot showed decreased protein expression of EMT markers Slug, Vimentin, and increased expression of E-cadherin (Fig. 5d). We examined the functional dependency of ZNF471 on TFAP2A by knocking down both TFAP2A and ZNF471 in GES1 and MKN1 cells. As shown in Fig. 5e, enhanced gastric cancer cell colony formation ability due to ZNF471 knockdown by shRNA was abolished through further knocking down of TFAP2A, indicating that the tumor-suppressive effect of ZNF471 is partially dependent on TFAP2A suppression.

Similarly, knocking down PLS3 in gastric cancer cells, significantly attenuated cell migration (Fig. 5f) and invasion ability (Fig. 5g) of BGC823 and MKN74 cells (Figure S4D). Western blot results validated the downregulation of Slug and elevation of E-Cadherin upon PLS3 knockdown (Fig. 5h). However, the cell proliferation ability was not affected by PLS3 (Figure S4E). Collectively, these results suggested that the tumor-suppressive effects of ZNF471 on cell proliferation, migration, and invasion are associated with the transcriptional suppression of its downstream target TFAP2A and PLS3.

### ZNF471 transcriptionally represses downstream target genes through recruiting KAP1

**Fig. 6:**
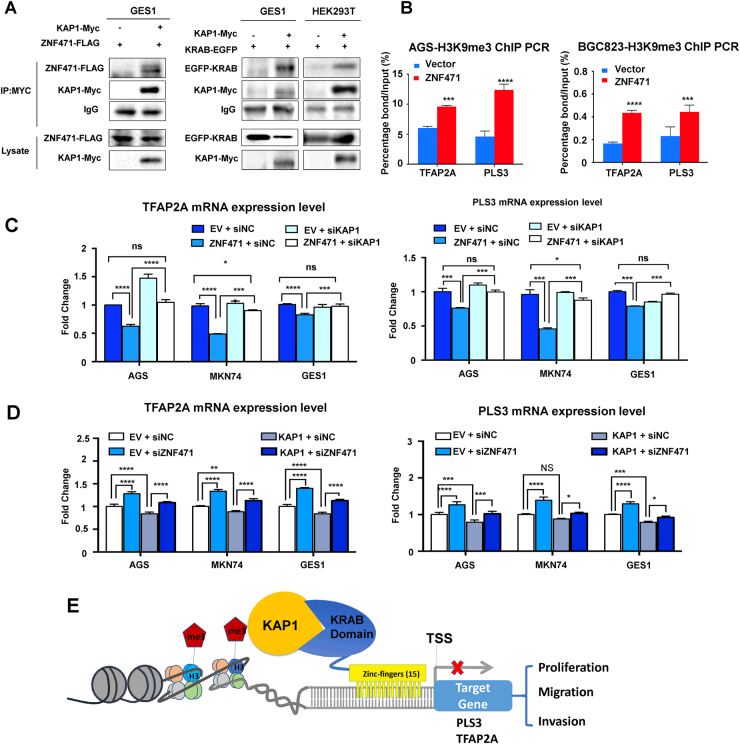
ZNF471 functioned as a transcriptional repressor in presence of co-repressor KAP1. **a** Co-immunoprecipitation assay with anti-Myc in GES1 cells expressing Flag-tagged full length ZNF471 or EGFP-tagged KRAB-domain of ZNF471, followed by Western blot with antibodies against the Flag-tagged ZNF471 or EGFP-tagged KRAB-domain. **b** Detection of transcription suppression histone marker H3K9me3 enrichment in the promoter region of TFAP2A and PLS3 by qPCR followed by chromatin immunoprecipitation (ChIP) with anti-H3K9me3 antibody. **c** Evaluation of transcriptional repression dependency on KAP1. The altered expression of downstream TFAP2A and PLS3 was quantified by qPCR, following KAP1 knock-downed by siRNA in ZNF471 overexpression cells. **d** KAP1-induced transcriptional repression on downstream genes depended on ZNF471. The altered expression of downstream TFAP2A and PLS3 was quantified by qPCR, following ZNF471 knock-downed by siRNA in KAP1 overexpression cells. **e** The molecular model depicted the mechanism of ZNF471 as a tumor suppressor in cooperation with KAP1 in gastric cancer. Data are expressed as mean ± S.D. *P < 0.05, **P < 0.01, ***P < 0.001, ****P < 0.0001

ZNF471 protein harbors a KRAB-domain at N terminal, which functions as a strong transcriptional repressor [10, 11, 17]. To mediate transcriptional repression at a target promoter, KRAB-domain requires KRAB-Interacting Protein 1 (KAP1) as a scaffold to recruit heterochromatin protein 1 isoforms (HP1 proteins), histone deacetylases (HDACs), and SET Domain Bifurcated 1 (SETDB1) to form a facultative heterochromatin environment [17–20]. However, it is still unknown whether KAP1 is a co-repressor of ZNF471. To investigate whether ZNF471 regulates downstream targets through recruiting KAP1 to the KRAB-domain at N terminal of the promoter region, we overexpressed full length ZNF471 plasmid with N terminal Flag-tag and Myc-tagged KAP1 in GES1 cell line. After immunoprecipitation using antibody against Myc-tag, flag-tagged ZNF471 was detected with anti-Flag antibody, indicating an association between ZNF471 and KAP1 (Fig. 6a). To specify the binding domain, we cloned the KRAB-domain of ZNF471 fused with GFP-tag and co-expresses with KAP1 in GES1. The detection of KRAB-domain with antibody against GFP indicated that ZNF471 recruited KAP1 through the KRAB-domain (Fig. 6a). The association between KRAB-domain of ZNF471 and KAP1 was also detected in HEK293T cells (Fig. 6a), indicating that KAP1 was involved in transcriptional repression of ZNF471.

Then we tested the effect of ZNF471 on the levels of H3K9me3, a critical epigenetic marker of transcriptional repression [10, 17], in the promoter region of TFAP2A and PLS3 by quantitative ChIP-PCR. As shown in Fig. 6b, H3K9me3 levels were significantly increased in the promoter region of TFAP2A and PLS3 in AGS and BGC823 cells transfected with ZNF471 compared to controls.

To evaluate whether ZNF471 repression of TFAP2A and PLS3 transcription depends on co-repressor KAP1, we examined TFAP2A and PLS3 mRNA expression in ZNF471 overexpressing AGS, MKN74 and GES1 cells with KAP1 knockdown. Knockdown of KAP1 in these cells restored expression of TFAP2A and PLS3 suppressed by ZNF471 (Fig. 6c, Figure S4F), suggesting that the transcriptional suppression of TFAP2A and PLS3 by ZNF471 was at least dependent on promoter recruitment of KAP1. Correspondingly, ZNF471 knockdown abolished the suppressive effect of KAP1 overexpression on TFAP2A and PLS3, indicating that KAP1 depends on ZNF471 to induce the transcription repression of downstream genes (Fig. 6d). Taken together, KAP1 serves as a co-repressor for ZNF471 in gastric cancer, inducing elevated H3K9me3 in the promoter of TFAP2A and PLS3 (Fig. 6e).

## Discussion

In this study, we identified ZNF471, a transcription factor, as a novel gene inactivated due promoter methylation. We found that the mRNA expression of ZNF471 was downregulated in 15 out of 16 gastric cancer cell lines (Fig. 1a), and the silenced ZNF471 expression was subsequently restored following treatment with a DNA methyltransferase activity inhibitor (Fig. 1d). The decreased mRNA expression of ZNF471 was then confirmed by BGS (Fig. 1c). In paired primary gastric cancer samples from patients, the promoter methylation status of ZNF471 by BGS was significantly higher in tumor than adjacent normal tissues (Fig. 1e). To ascertain the clinical importance of ZNF471, we analyzed 120 gastric cancer patients for the methylation status of an 8-CpG-site region in ZNF471 promoter. Multivariate analysis revealed that methylation status of 8-CpG-site median value and individual CpG sites (especially CpG3) functions as an independent prognosticator for overall survival of gastric cancer patients (Fig. 1h). Single-specific CpG-site in the relevance of predictive of prognosis has recently been supported in various cancers. In chronic lymphatic leukemia, a single CpG dinucleotide has been identified to be important for ZAP-70 expression and prognosis [21]. In soft-tissue sarcomas, CpG107 methylation status of the PLAGL1 P1 promoter was the first prognostic biomarker for both metastasis-free survival and overall survival in the US [22]. In gastric cancer, methylation status of one or more individual CpG sites in genes DACT1, PAX5, and RUNX3 promoter region was also applicable for prognosis [23–25]. Thus, these data highlight the potential of individual CpG sites of ZNF471 as a clinical prognostic marker in gastric cancer.

A series of in vitro and in vivo functional experiments revealed that ZNF471 possesses a tumor-suppressive function in gastric cancer. Ectopic expression of ZNF471 in silenced or low-expression gastric cancer cell lines (AGS, BGC823, and MKN74) inhibited cell proliferation and colony formation, induced apoptosis and arrested cells at G0/G1 phase (Fig. 2h). Increased apoptosis due to ZNF471 overexpression was evidenced by activation of the caspase markers. G1 arrest by ZNF471 was associated with the suppression of cyclin D1 and CDK4 as well as the induction of p27 and p21. The cyclin D1/CDK4 complex is a key regulator for G1/S checkpoint in cell cycle, and p27 and p21are two key CDK negative regulators [26]. Moreover, the re-expression of ZNF471 in gastric cancer cell lines (BGC823 and MKN74) significantly inhibited their migration and invasion abilities. The molecular mechanisms by which ZNF471 exerts its anti-invasive function were shown to be via enhancing E-cadherin expression and inhibiting Slug and Vimentin expression. E-cadherin functions as an invasion suppressor; whereas Slug and Vimentin promote cell motility and invasion in cancers [27, 28]. Taken together, our results indicated for the first time that ZNF471 functions as a tumor suppressor in gastric cancer.

Zinc-finger proteins compose the largest family of sequence-specific DNA binding proteins among all the transcription factors in eukaryotic cells, exhibiting great diversity of biological functions [29]. Thus, identifying the target genes regulated by ZNF471 will be important for understanding the molecular basis of its tumor-suppressive effect. The DNA binding motif model of ZNF471 was computed based on the protein sequence from UniProt database, and was applied for the genomic locations scanning of the predicted binding sites. We narrowed down the binding targets to 6 genes using the genomic regulation database, which were validated by ChIP-PCR in AGS and BGC823. The mRNA of 6 genes were evaluated for transcriptional activity of ZNF471, and the mRNA levels of TFAP2A, PLS3 and PSMB8 were consistently suppressed in AGS and BGC with ZNF471 overexpression (Fig. 4). EMSA experiment further validated the binding activity and specificity of ZNF471 to the promoter of TFAP2A and PLS3 (Fig. 4). Combining the EMSA result and GSEA with TCGA Stomach Cancer gene expression RNAseq data, we identified TFAP2A and PLS3, as the critical targets of ZNF471. We demonstrated the oncogenic properties of TFAP2A in promoting cell proliferation, migration and invasion and PLS3 in inducing cell migration and invasion in gastric cancer cell lines (Fig. 5). It was reported that TFAP2A had oncogenic roles in other cancer types of head and neck, breast and nasopharyngeal carcinoma [12, 30, 31]. The oncogenic role of PLS3 on EMT was also reported in colorectal cancer [32]. Collectively, these findings suggested that ZNF471 is a novel gastric tumor suppressor which functions by transcriptional inhibition of its oncogenic targets, TFAP2A and PLS3.

KRAB-domain generally acts as a strong repressor, since it recruits a bunch of co-repressors to form a complex [10]. Among the co-repressors, KAP1 is the crucial protein that functions as a scaffold for the binding of other co-factors, mainly the repressors. We identified the interaction between KRAB-domain of ZNF471 and KAP1 by Co-IP assay (Fig. 6a), which also has been validated by other KRAB-ZFPs [18]. We demonstrated that the downstream genes TFAP2A and PLS3 were transcriptionally repressed by ZNF471. We found that the histone mark H3K9me3 was enriched at the promoter region of TFAP2A and PLS3, which is strongly associated with KRAB-ZFP recruited KAP1 co-repressor complex [33] (Fig. 6b). Hence, ZNF471 suppressed gastric cancer progression by transcriptionally repressing its downstream targets TFAP2A and PLS3 in co-operating with KAP1.

ZNF471 is a transcription factor preferentially silenced by DNA methylation in gastric cancer, thus the dysregulation of ZNF471 may affect multiple cellular processes involved in its transcriptional regulation. Therefore, future research could focus on identification of the promoter CpG sites, whose methylation directly contribute to the biological behaviors of ZNF471 and serve as potential therapeutic targets. Once the functional CpG sites are validated, the CRISPR/Cas9 based DNA methylation editing tool can be applied to predictably alter the epigenetic state-of-target sequences [34], thereby fostering the development of targeted cancer therapy.

In summary, ZNF471 is a novel tumor suppressor in gastric cancer, inhibiting cell growth, triggering apoptosis and cell cycle arrest as well as impairing cell migration and invasion abilities. The tumor-suppressive function of ZNF471 was mediated by directly inhibiting its downstream targets TFAP2A and PLS3 transcription. KAP1 is a co-suppressor of ZNF471. Methylation status of 8-CpG-site median value and individual CpG-site (CpG3) are independent prognosticators for overall survival of gastric cancer patients.

## Materials and methods

### Cell lines

AGS, Kato III, and NCI-N87 cell lines were ordered from American Type Culture Collection (ATCC, Manassas, VA; 2014). MKN1, MKN45, MKN74, and TMK1 were obtained from Japanese Collection of Research Bioresources Cell Bank (JCRB, Japan; 2014). SNU16, SNU638, SNU668, SNU719, and YCC10 were purchased from the Korean Cell Line Bank (Seoul, Korea; 2015). BGC823, GES1, HEK293T, HGC27, MGC803, and SGC7901 were from Cell bank of Chinese Academy of Sciences (Shanghai, China; 2015). All cell lines were authenticated and tested negative for mycoplasma contamination by the providers. All experiments were performed using cells with passage number <10 after thawing. Cell lines were maintained in RPMI 1640 or Dulbecco’s modified Eagle’s medium (DMEM) (Gibco BRL, Grand Island, NY) with 10% fetal bovine serum and 1% Penicillin–Streptomycin–Glutamine (Gibco BRL). The adult normal human stomach RNA was purchased from Clontech (Takara Bio USA, Mountain View, CA).

### Human gastric specimens

Gastric tumor tissues of 120 were collected from surgical resection center of the Beijing Cancer Hospital and Institute, Beijing, China. Twenty-eight paired stomach tumor and tumor adjacent samples were collected during surgical resection, whereas five biopsies of normal gastric mucosa from healthy people were obtained from endoscopy center at the Prince of Wales Hospital, The Chinese University of Hong Kong, Hong Kong. The sample collection was approved by the Clinical Research Ethics Committee of both Beijing Cancer Hospital and Institute and the Chinese University of Hong Kong.

### Plasmids, small interfering RNA (siRNA) and short hairpin RNA (shRNA)

ZNF471 overexpression plasmid was purchased from Inovogen (Beijing, China). ZNF471 overexpression plasmid was purchased from Inovogen (Beijing, China). After transfection, ZNF471-expressing cell pool was selected with G418 (Calbiochem, Darmstadt, Germany) for 3–5 days, with G481 and full medium refreshed every day. The stable pool was stocked in aliquots after the overexpression level was confirmed by Western blot and PCR. Expression plasmid was pcDNA3.1(+) (Invitrogen, Carlsbad, California), not inducible promoter. The shZNF471 vector (synthesized from siZNF471-1), siRNAs against ZNF471, TFAP2A, PLS3, and KAP1 were obtained from GenePharma (Shanghai, China). The sequence for siKAP1 is adopted from published article [35], and detailed sequence information was attached in the table as supplementary (Supplementary Table S5).

### Bisulfite modification of DNA and BGS

Genome DNA bisulfite modification was performed with kit from Zymo Research (Irvine, CA). Following amplification of mDNA with designed BGS primers for 40-45 PCR cycles, the PCR products were sequenced with Sanger method. Each sample was sequenced once and calculated for the methylation percentage as described previously [36]. The region for ZNF471 methylation analysis was chosen with the following criteria: (a) within a CpG island; (b) with high CG percentage; (c) significant methylation value difference between gastric cancer tissues and adjacent non-cancer tissues; (d) correlated with mRNA expression level. Eight CpG sites range from −74 to +30 relative to the first exon were evaluated. The primer sequences are as follows: forward: GAGGATAATTTTTTTAATATTAGGTTA; reverse: TACCCTTCCTAACCCAA.

### Demethylation treatment

Cells were seeded at a density of 1 × 10^5^ cells/ml in 6-well plate and grew for 24 h. Cells were then treated with 2 μM 5-Aza (Sigma-Aldrich, St Louis, MO, USA) for 96 h, with drug and medium refreshed every day. Cells were then harvested and gene expression was analyzed using RT-PCR.

### Fluorescent immunohistochemistry

AGS and BGC823 cells were seeded on coverslips on a six-well plate at 60% confluency and transfected with pcDNA3.1-Flag-ZNF471. After transfection for 48 h, cells were fixed with 4% paraformaldehyde, followed by permeabilization with 0.3% Triton X-100. The fixed cells were then blocked with 3% bovine serum albumin, and stained with monoclonal antibody to Flag M2 (1:50 dilution, F1804) (Sigma-Aldrich, St Louis, Missouri, MO) overnight at 4 °C. Following the incubation with Alexa Fluor® 546 conjugate goat anti-rabbit IgG (1:500 dilution ratio) (Life technologies), cells were preserved with ProLong® Gold Antifade Mountant with DAPI (Life technologies). Images were subsequently captured by confocal microscope.

### Cell growth curve and colony formation assay

Stable ZNF471-expressing gastric cancer cells (AGS, BGC823, and MKN74) and cells with empty pcDNA3.1 were seeded to 96-well plates (1000 cell/well), then 3-(4,5-dimethylthiazol-2-yl)-2,5-diphenyltetrazolium bromide (MTT; Sigma-Aldrich) was added to each well to determine the cell viability. The cell growth curve was plotted with data collected from six continuous time points. To perform the cell colony formation assay, equal number of cells was seeded to 6-well plate and then colonies were fixed with 70% ethanol and stained with 5% crystal violet following 10–14 days’ incubation. Stained colonies (≥ 50 cells/colony) were numbered for analysis.

### Apoptosis and cell cycle determination

Cell apoptosis was stained by Annexin-phycoerythrin and 7-aminoactinomycin kit (Becton Dickinson Biosciences, San Jose, CA). Cell cycle was analyzed as previous described [37]. BD Accuri C6 (Becton Dickinson Biosciences), FlowJo (Version OSX 10.6; Ashland, Oregon;) and ModFit LT (version 4) were applied for data collection and analysis.

### Subcutaneous xenograft mouse models

BGC823 cells stably expressing control vector or ZNF471 (5 × 10^6^) cells in 0.1 ml phosphate-buffered saline (PBS) were injected subcutaneously into the left and right dorsal flank, respectively, of 4-week-old male Balb/c nude mice (*n* = 5). Tumor size was measured every 2 days using a digital caliper. All mice were sacrificed for harvesting tumors 14 weeks after tumor cell injection. Tumor volume was calculated by the formula: tumor volume [mm^3^] = (length [mm]) × (width [mm])^2^ × 0.5. All experimental procedures were approved by the Animal Ethics Committee of the Chinese University of Hong Kong.

### Binding motif prediction

The protein sequence of ZNF471 was downloaded from the UniProt database (http://www.uniprot.org/uniprot/Q9BX82) and was then inputted into the state-of-the-art zinc-finger DNA motif recognition model [13] to calculate the DNA binding motif model of ZNF471, i.e., Position Weight Matrix (PWM) model. After that, the DNA binding motif model was inputted to the FIMO online software [38], which scanned on the whole human genome (hg19) for the genomic locations of the predicted binding sites of ZNF471. The list of the predicted genomic locations was outputted for matching the genes transcriptionally regulated by ZNF471.

### Chromatin immunoprecipitation (ChIP) PCR

ChIP experiments followed previous described protocol [39]. Briefly, stable ZNF471-expressing AGS or BGC823 cells and vector-expressing cells were cross-linked with formaldehyde collected for protein extraction. Chromatin from extracted cross-linked nuclei was sheared to 200-1000 bp by sonication and precipitated with antibodies to Flag (#14793; Cell Signaling Technology, Danvers, MA) or Histone H3 tri methyl Lysine 9 residue (H3K9me3, ab8898; Cambridge, MA). The same amount of non-specific IgG (SC-2027×) (Santa Cruz, Dallas, TX) was used as control.; Immunoprecipitated protein-DNA complex was then captured with protein-G magnetic beads (16-662; Millipore, Billerica, MA), after that immunoprecipitated DNA was purified with kit (Qiagen, Valencia, CA). Enrichment of downstream genes was examined by both real-time PCR and conventional PCR, and primer sequences are attached in the Supplementary Table S6. The enrichment of ZNF471 binding to the promoter of downstream genes was calculated by percent input method. The experiment was repeated three times independently.

### Electrophoretic mobility shift assay (EMSA)

Nuclear extracts were prepared from 293 T cells transfected with pCDNA3.1-ZNF471 for 48 h with NE-PER Nuclear and Cytoplasmic extraction kit (Thermo Fisher Scientific). EMSA was performed according to the manual provided by LightShift™ Chemiluminescent EMSA Kit (Thermo Fisher Scientific). In brief, the biotin-labeled 42-bp dsDNA was prepared by annealing two complementary HPLC-purified DNA oligosin annealing buffer (10 mM Tris, 1 mM EDTA, 50 mM NaCl, pH 8) at a concentration of 1 pmol/µl in a temperature gradient of 0.1 °C/s from 95 °C to 26 °C. The EMSA reaction was prepared with 50 fmol DNA probe, purified protein and 50 ng/μl Poly (dΙ•dC) in binding buffer (10 mM Tris, 50 mM KCl, 1 mM DTT; pH 7.5). After 20 min incubation, free DNA and DNA–protein complexes were mixed with loading dye and resolved by electrophoresis for 45 min at 100 V. The specimens were transferred onto a 0.45-μm Biodyne B nylon membrane (Thermo Fisher Scientific) at 380 mA for 30 min at 4 °C, and cross-linked to the membrane using a TL-2000 UV Translinker (Ultra-Violet Products). The blots were developed using Chemiluminescent Nucleic Acid Detection Module Kit (Thermo Scientific).

TFAP2A:

5′-GTGAAAGAGAAAGAGGCAGAGAGGGAGACCGAAGGAGAGAGC-3′,

5′-GCTCTCTCCTTCGGTCTCCCTCTCTGCCTCTTTCTCTTTCAC-3′;

PLS3:

5′-TGCAAAGATTCCGAGGTGCAGAAGTTGTCTGAGTGCGTTGGT-3′,

5′-ACCAACGCACTCAGACAACTTCTGCACCTCGGAATCTTTGCA-3′

PSMB8:

5′-CACTTCCTCCTCCGAGAGCGGACAGATCTCTGGGTGCTGGGC-3′

5’- GCCCAGCACCCAGAGATCTGTCCGCTCTCGGAGGAGGAAGTG -3’

### Co-immunoprecipitation (Co-IP)

Ges1 cells were co-transfected with pcDNA3.1-Flag-ZNF471, and /or pcDNA3.1-Myc-KAP1, pEGFP-C1-HP1β (green fluorescent protein (GFP)-tagged mouse HP1b). After 48 h of transfection, total protein was extracted in RIPA buffer (5% sodium deoxy cholate, 0.1% SDS, 10 mm Na_3_VO_4_, 50 mM NaF, 1% Nonidet P40, phosphate-buffered saline buffer, 13 protease inhibitor mixture) for 1 h on ice, followed by sonication of five 30 s/30 s cycles. Cell lysates were centrifuged and the pellets discarded. Anti-Myc antibody (Santa Cruz Biotechnology) or Anti-Flag antibody (F1804; Sigma-Aldrich) of 2 μg and 50 ml protein-G Mega beads (Millipore) were added to 1 μg protein lysate for overnight incubation at 4 °C. Finally, immunoprecipitated protein complex was added with 2×loading buffer and boiled to denature the protein and separate it from protein-G beads. The precipitated proteins were evaluated by Western blot using primary antibody to GFP (Santa Cruz).

### Statistical analysis

All the results were expressed as mean ± S.D. Student *t* test or Mann–Whitney *U* test was used for comparing means between two groups. Two-way analysis of variance was used for comparison of differences between growth curves. For clinical data, statistical analysis was performed using the SPSS software (version 16.0, SPSS Inc, Chicago, IL, 2007). Univariate and multivariate Cox regression analysis was performed to assess the prognostic value of ZNF471 methylation. Overall survival in relation to methylation status was evaluated from Kaplan–Meier survival curves and the log-rank test. *P* *<* 0.05 was taken as statistical significance.

## Electronic supplementary material

Supplementary Table 1-7(DOCX 56 kb)

Figure S1(TIF 1141 kb)

Figure S2(TIF 137 kb)

Figure S3(TIF 55 kb)

Figure S4(TIF 344 kb)

Figure S5(TIF 199 kb)

Supplementary Information 1(DOCX 26 kb)

Supplementary Information 2(PDF 2156 kb)
