# Supplementary material for: Zinc-finger protein 471 suppresses gastric cancer through transcriptionally repressing downstream oncogenic PLS3 and TFAP2A
Source: Oncogene. 2018 Apr 3;37(26):3601–16. doi: 10.1038/s41388-018-0220-5 (PMC6021371; doi:10.1038/s41388-018-0220-5)
Supplement: Supplementary file 1 — Supplementary Table 1-7(DOCX 56 kb) [file 41388_2018_220_MOESM1_ESM.docx]

**Supplementary Table 1. General information of patients with gastric cancer from Beijing Cancer Hospital & Institute**

| **Viable** | **Total number** |
| --- | --- |
| Gender |  |
| Male | 81 |
| Female | 39 |
| Age |  |
| <65 | 72 |
| >=65 | 48 |
| Stage |  |
| I/II | 35 |
| III/IV | 85 |
| Differentiation |  |
| High | 3 |
| Medium | 62 |
| Low | 55 |
| Vital status |  |
| Died | 58 |
| Survival | 62 |

**Supplementary Table 2. Correlation of ZNF471 CpG site3 methylation level with clinical pathological features of gastric cancer patients.**

| **Viable** | **High Methylation** | **Low Methylation** | ***P* value**  (χ2 test) |
| --- | --- | --- | --- |
| Age |  |  | 0.646 |
| <65 | 16 | 56 |  |
| >=65 | 9 | 39 |  |
| Gender |  |  | 0.308 |
| Male | 19 | 62 |  |
| Female | 6 | 33 |  |
| Lauren |  |  | 0.482 |
| Intestinal | 3 | 17 |  |
| Diffuse | 22 | 78 |  |
| TNM Stage |  |  | 0.104 |
| I/II | 4 | 31 |  |
| III/IV | 21 | 65 |  |
| Size of Tumour |  |  | 0.228 |
| T1+T2+T3 | 2 | 17 |  |
| T4 | 23 | 78 |  |
| Nodal status |  |  | 0.3817 |
| N0 | 4 | 23 |  |
| N1-N3 | 21 | 72 |  |
| Distant metastases |  |  | **0.0039** |
| M0 | 19 | 90 |  |
| M1 | 6 | 5 |  |

**Supplementary Table 3. The list of 125 cell lines used in DNase-seq form ECODE**

| cell | Tissue | Karyotype | | Sex | Vendor ID |
| --- | --- | --- | --- | --- | --- |
| 8988T | liver | cancer | | F | [DSMZ ACC 162](http://www.dsmz.de/human_and_animal_cell_lines/info.php?dsmz_nr=162&term=162&highlight=) |
| A549 | epithelium | cancer | | M | [ATCC CCL-185](http://www.atcc.org/ATCCAdvancedCatalogSearch/ProductDetails/tabid/452/Default.aspx?ATCCNum=CCL-185&Template=cellBiology) |
| Adult_CD4_Th0 | blood | normal | | B | [Dr. David Randolph at University of Alabama, Birmingham Adult_CD4_Th0](http://138.26.61.118/depts/MEB/SOMResearchFaculty/currentfacultydata.asp?ID=DRDRDR) |
| AG04449 | skin | normal | | M | [Coriell AG04449](http://ccr.coriell.org/Sections/Search/Sample_Detail.aspx?Ref=AG04449) |
| AG04450 | lung | normal | | M | [Coriell AG04450](http://ccr.coriell.org/Sections/Search/Sample_Detail.aspx?Ref=AG04450) |
| AG09309 | skin |  | | F | [Coriell AG09309](http://ccr.coriell.org/Sections/Search/Sample_Detail.aspx?Ref=AG09309) |
| AG09319 | gingival | normal | | F | [Coriell AG09319](http://ccr.coriell.org/Sections/Search/Sample_Detail.aspx?Ref=AG09319) |
| AG10803 | skin |  | | M | [Coriell AG10803](http://ccr.coriell.org/Sections/Search/Sample_Detail.aspx?Ref=AG10803) |
| AoAF | blood vessel | normal | | F | [Lonza CC-7014, CC-7014T75](http://www.lonza.com/) |
| AoSMC | blood vessel | normal | | U | [Lonza CC-2571](http://www.lonza.com/) |
| BE2_C | brain | cancer | | M | [ATCC CRL-2268](http://www.atcc.org/ATCCAdvancedCatalogSearch/ProductDetails/tabid/452/Default.aspx?ATCCNum=CRL-2268&Template=cellBiology) |
| BJ | skin | normal | | M | [ATCC CRL-2522](http://www.atcc.org/ATCCAdvancedCatalogSearch/ProductDetails/tabid/452/Default.aspx?ATCCNum=CRL-2522&Template=cellBiology) |
| Caco-2 | colon | cancer | | M | [ATCC HTB-37](http://www.atcc.org/ATCCAdvancedCatalogSearch/ProductDetails/tabid/452/Default.aspx?ATCCNum=HTB-37&Template=cellBiology) |
| CD20+ | blood | normal | | F | [Dr. Heimfeld Lab at FHCRC CD20+](http://labs.fhcrc.org/heimfeld/index.html) |
| CD34+_Mobilized | blood |  | | M | [Dr. Heimfeld Lab at FHCRC FHCRC](http://labs.fhcrc.org/heimfeld/index.html) |
| Chorion | fetal membrane |  | | U | [Dr. Amy Murtha at Duke University](http://www.dukehealth.org/physicians/amy_p_murtha) |
| CLL | blood | cancer | | F | [Dr. Jennifer Brown at Harvard Medical School. CLL](http://www.dfhcc.harvard.edu/membership/profile/member/669/0/) |
| CMK | blood | cancer | | M | [DSMZ ACC-392](http://www.dsmz.de/human_and_animal_cell_lines/info.php?dsmz_nr=392&from=cell_line_index&select=search_for_term&term=CMK&preselect=human;hamster;mouse;rat;insect;other&firstload=0) |
| Fibrobl | skin | normal | | F | [Coriell AG08470](http://ccr.coriell.org/Sections/Search/Sample_Detail.aspx?Ref=AG08470&PgId=166) |
| FibroP | skin | normal | | U | [Paul Tesar at Case Western University FibroP](http://genetics.case.edu/page.php?page_id=5&LN=Tesar&FN=Paul) |
| Gliobla | brain | cancer | | U | [Dr. Darrell Bigner at Duke University Medical Center D54](http://www.cancer.duke.edu/btc/modules/facultystaff1/index.php?id=85) |
| GM06990 | blood |  | | F | [Coriell GM06990](http://ccr.coriell.org/Sections/Search/Sample_Detail.aspx?Ref=GM06990&PgId=166) |
| GM12864 | blood |  | | M | [Coriell GM12864](http://ccr.coriell.org/Sections/Search/Sample_Detail.aspx?Ref=GM12864&PgId=166) |
| GM12865 | blood |  | | F | [Coriell GM12865](http://ccr.coriell.org/sections/Search/Sample_Detail.aspx?Ref=GM12865&PgId=166) |
| GM12878 | blood | normal | | F | [Coriell GM12878](http://ccr.coriell.org/Sections/Search/Sample_Detail.aspx?Ref=GM12878&PgId=166) |
| GM12891 | blood |  | | M | [Coriell GM12891](http://ccr.coriell.org/Sections/Search/Sample_Detail.aspx?Ref=GM12891) |
| GM12892 | blood |  | | F | [Coriell GM12892](http://ccr.coriell.org/Sections/Search/Sample_Detail.aspx?Ref=GM12892) |
| GM18507 | blood |  | | M | [Coriell GM18507](http://ccr.coriell.org/Sections/Search/Sample_Detail.aspx?Ref=GM18507) |
| GM19238 | blood |  | | F | [Coriell GM19238](http://ccr.coriell.org/Sections/Search/Sample_Detail.aspx?Ref=GM19238) |
| GM19239 | blood |  | | M | [Coriell GM19239](http://ccr.coriell.org/Sections/Search/Sample_Detail.aspx?Ref=GM19239) |
| GM19240 | blood |  | | F | [Coriell GM19240](http://ccr.coriell.org/Sections/Search/Sample_Detail.aspx?Ref=GM19240) |
| H1-hESC | embryonic stem cell | normal | | M | [WiCell Research Institute WA01](https://ordering.wicell.org/index.php?option=com_oscommerce&Itemid=192) |
| H7-hESC |  |  | | U | [WiCell Research Institute WA07 (H7)](http://www.wicell.org/index.php) |
| H9ES | embryonic stem cell |  | | F | [WiCell Research Institute WA09 (H9)](http://www.wicell.org/index.php) |
| HA-h | brain hippocampus | normal | | U | [ScienCell 1830](http://www.sciencellonline.com/human-astrocytes-hippocampal.html) |
| HA-sp | spinal cord | normal | | U | [ScienCell 1820](http://www.sciencellonline.com/human-astrocytes-spinal-cord.html) |
| HAc | cerebellar | normal | | U | [ScienCell 1810](http://www.sciencellonline.com/human-astrocytes-cerebellar.html) |
| HAEpiC | epithelium | normal | | U | [ScienCell 7110](http://www.sciencellonline.com/haepic.html) |
| HBMEC | blood vessel | normal | | U | [ScienCell 1000](http://www.sciencellonline.com/human-brain-microvascular-endothelial-cells.html) |
| HCF | heart | normal | | U | [ScienCell 6300](http://www.sciencellonline.com/human-cardiac-fibroblasts.html) |
| HCFaa | heart | normal | | F | [ScienCell 6320](http://www.sciencellonline.com/human-cardiac-fibroblasts-adult-atrial.html) |
| HCM | heart | normal | | U | [ScienCell 6200](http://www.sciencellonline.com/human-cardiac-myocytes.html) |
| HConF | eye |  | | U | [ScienCell 6570](http://www.sciencellonline.com/hconf.html) |
| HCPEpiC | epithelium | normal | | U | [ScienCell 1310](http://www.sciencellonline.com/human-choroid-plexus-epithelial-cells.html) |
| HCT-116 | colon | cancer | | M | [ATCC CCL-247](http://www.atcc.org/ATCCAdvancedCatalogSearch/ProductDetails/tabid/452/Default.aspx?ATCCNum=CCL-247&Template=cellBiology) |
| HEEpiC | epithelium | normal | | U | [ScienCell 2720](http://www.sciencellonline.com/human-esophageal-epithelial-cells.html) |
| HeLa-S3 | cervix | cancer | | F | [ATCC CCL-2.2](http://www.atcc.org/ATCCAdvancedCatalogSearch/ProductDetails/tabid/452/Default.aspx?ATCCNum=CCL-2.2&Template=cellBiology) |
| Hepatocytes | liver | normal | | B | [zen-bio Missing](http://www.zen-bio.com/) |
| HepG2 | liver | cancer | | M | [ATCC HB-8065](http://www.atcc.org/ATCCAdvancedCatalogSearch/ProductDetails/tabid/452/Default.aspx?ATCCNum=HB-8065&Template=cellBiology) |
| HFF | foreskin | normal | | M | [Dr. Beverly Torok-Storb at Fred Hutchison Cancer Research Center HFF](http://myprofile.cos.com/torokstorb) |
| HFF-Myc | foreskin | normal | | M | [Dr. Beverly Torok-Storb at Fred Hutchison Cancer Research Center HFF-Myc](http://myprofile.cos.com/torokstorb) |
| HGF | gingiva | normal | | U | [ScienCell 2620](http://www.sciencellonline.com/human-gingival-fibroblasts.html) |
| HIPEpiC | epithelium | normal | | U | [ScienCell 6560](http://www.sciencellonline.com/hipepic.html) |
| HL-60 | blood | cancer | | F | [ATCC CCL-240](http://www.atcc.org/ATCCAdvancedCatalogSearch/ProductDetails/tabid/452/Default.aspx?ATCCNum=CCL-240&Template=cellBiology) |
| HMEC | breast | normal | | U | [Lonza CC-2551](http://www.lonza.com/) |
| HMF | mammary |  | | F | [ScienCell 7630](http://www.sciencellonline.com/hmf.html) |
| HMVEC-dAd | blood vessel | normal | | F | [Lonza CC-2543,CC-3202](http://www.lonza.com/) |
| HMVEC-dBl-Ad | blood vessel | normal | | F | [Lonza CC-2811, CC-2811T75](http://www.lonza.com/) |
| HMVEC-dBl-Neo | blood vessel | normal | | M | [Lonza CC-2813, CC-2813T75](http://www.lonza.com/) |
| HMVEC-dLy-Ad | blood vessel | normal | | F | [Lonza CC-2810, CC-2810T75](http://www.lonza.com/) |
| HMVEC-dLy-Neo | blood vessel | normal | | M | [Lonza CC-2812, CC-2812T25](http://www.lonza.com/) |
| HMVEC-dNeo | blood vessel | normal | | M | [Lonza CC-2505, CC-2505T225](http://www.lonza.com/) |
| HMVEC-LBl | blood vessel | normal | | F | [Lonza CC-2815, CC-2815T75](http://www.lonza.com/) |
| HMVEC-LLy | blood vessel | normal | | F | [Lonza CC-2814, CC-2814T25](http://www.lonza.com/) |
| HNPCEpiC | epithelium | normal | | U | [ScienCell 6580](http://www.sciencellonline.com/hnpcepic.html) |
| HPAEC | blood vessel | normal | | F | [Lonza CC-2530,CC-3162](http://www.lonza.com/) |
| HPAF | blood vessel | normal | | U | [ScienCell 3120](http://www.sciencellonline.com/hpaaf.html) |
| HPDE6-E6E7 | pancreatic duct | normal | | F | [Dr. Ming-Sound Tsao, Ontario Cancer Institute HPDE6-E6E7](http://www.uhnresearch.ca/researchers/profile.php?lookup=6118) |
| HPdLF | epithelium | normal | | M | [Lonza CC-7049](http://www.lonza.com/) |
| HPF | lung | normal | | U | [ScienCell 3300](http://www.sciencellonline.com/hpf.html) |
| HRCEpiC | epithelium | normal | | U | [Lonza CC-2554](http://www.lonza.com/) |
| HRE | epithelium | normal | | U | [Lonza CC-2556](http://www.lonza.com/) |
| HRGEC | kidney | normal | | U | [ScienCell 4000](http://www.sciencellonline.com/human-renal-glomerular-endothelial-cells.html) |
| HRPEpiC | epithelium | normal | | U | [ScienCell 6540](http://www.sciencellonline.com/hrpepic.html) |
| HSMM | muscle | normal | | U | [Refer to the protocol documents for differing sources CC-2580](http://www.lonza.com/) |
| HSMM_emb | muscle |  | | U | [Duke/UNC/UT/EBI ENCODE group fetal myoblast cell strain (H246 and H275) from Dr Stephen Hauschka- U. of Washington 1980](http://depts.washington.edu/biowww/faculty/hauschka-steve/) |
| HSMMtube | muscle | normal | | U | [Lonza See HSMM](http://www.lonza.com/) |
| HTR8svn | blastula | normal | | F | [Charles H. Graham, Department of Anatomy & Cell Biology, Queen's University at Kingston, Kingston, Ontario, Canada HTR8svn](http://anatomy.queensu.ca/faculty/graham.cfm) |
| Huh-7 | liver | cancer | | M | [Dr. Ravi Jhaveri at Duke University Huh-7](http://www.dukehealth.org/physicians/ravi_r_jhaveri) |
| Huh-7.5 | liver | cancer | | M | [Dr. Ravi Jhaveri at Duke University Huh-7.5](http://www.dukehealth.org/physicians/ravi_r_jhaveri) |
| HUVEC | blood vessel | normal | | U | [Lonza CC-2517](http://www.lonza.com/) |
| HVMF | connective | normal | | U | [ScienCell 7130](http://www.sciencellonline.com/hvmf.html) |
| iPS | induced pluripotent stem cell | | | B | [Josh Chenoweth, Laboratory of Molecular Biology, National Institutes of Health, NINDS iPS](http://intra.ninds.nih.gov/) |
| Ishikawa | uterus | |  | F | [Sigma-Aldrich 99040201](http://www.sigmaaldrich.com/catalog/ProductDetail.do?D7=0&N5=SEARCH_CONCAT_PNO%7CBRAND_KEY&N4=99040201%7CSIGMA&N25=0&QS=ON&F=SPEC) |
| Jurkat | blood | | cancer | M | [ATCC TIB-152](http://www.atcc.org/ATCCAdvancedCatalogSearch/ProductDetails/tabid/452/Default.aspx?ATCCNum=TIB-152&Template=cellBiology) |
| K562 | blood | | cancer | F | [ATCC CCL-243](http://www.atcc.org/ATCCAdvancedCatalogSearch/ProductDetails/tabid/452/Default.aspx?ATCCNum=CCL-243&Template=cellBiology) |
| LNCaP | prostate | | cancer | M | [ATCC CRL-1740](http://www.atcc.org/ATCCAdvancedCatalogSearch/ProductDetails/tabid/452/Default.aspx?ATCCNum=CRL-1740&Template=cellBiology) |
| MCF-7 | breast | | cancer | F | [ATCC HTB-22](http://www.atcc.org/ATCCAdvancedCatalogSearch/ProductDetails/tabid/452/Default.aspx?ATCCNum=HTB-22&Template=cellBiology) |
| Medullo | brain | | cancer | U | [Dr. Darrell Bigner at Duke University Medical Center](http://www.cancer.duke.edu/btc/modules/facultystaff1/index.php?id=85) |
| Melano | skin | | normal | U | [ScienCell 2200](http://www.sciencellonline.com/human-epidermal-melanocytes-light.html) |
| Monocytes-CD14+_RO01746 | monocytes | | normal | F | [S. Heimfeld Laboratory, FHCRC Monocytes-CD14+_RO01746](http://labs.fhcrc.org/heimfeld/contact.html) |
| Myometr | myometrium | | normal | F | [Dr. Jennifer Condon of Magee Women's Research Institute](http://www.mwrif.org/188/condon-jeyasuria-lab) |
| NB4 | blood | | cancer | U | [National Cell Culture Center http://www.nccc.com/ NB4](http://www.dsmz.de/) |
| NH-A | brain | | normal | U | [Lonza CC-2565](http://www.lonza.com/) |
| NHDF-Ad | skin | | normal | F | [Lonza CC-2511, CC-2511T225](http://www.lonza.com/) |
| NHDF-neo | skin | | normal | U | [Lonza CC-2509](http://www.lonza.com/) |
| NHEK | skin | | normal | U | [Lonza CC-2501](http://www.lonza.com/) |
| NHLF | lung | | normal | U | [Lonza CC-2512](http://www.lonza.com/) |
| NT2-D1 | testis | | cancer | M | [ATCC CRL-1973](http://www.atcc.org/ATCCAdvancedCatalogSearch/ProductDetails/tabid/452/Default.aspx?ATCCNum=CRL-1973&Template=cellBiology) |
| Osteobl | bone | | normal | U | [Lonza CC-2538](http://www.lonza.com/) |
| PANC-1 | pancreas | | cancer | M | [ATCC CRL-1469](http://www.atcc.org/ATCCAdvancedCatalogSearch/ProductDetails/tabid/452/Default.aspx?ATCCNum=CRL-1469&Template=cellBiology) |
| PanIsletD | pancreas | |  | B | [National Disease Research Interchange PanIsletD](http://www.ndriresource.org/NDRI_Initiatives/Pancreatic_Islets/31/) |
| PanIslets | pancreas | | normal | M | [Refer to publication or NDRI PanIslets](http://www.ndriresource.org/NDRI_Initiatives/Pancreatic_Islets/31/) |
| pHTE | epithelium | |  | U | [Dr. Cal Cotton at Case Western Reserve University](https://www.case.edu/cgi-bin/directory/pb_lookup.cgi?search_text=calvin+cotton&submit.x=0&submit.y=0&submit=Search&search_method=regular&page_num=1) |
| PrEC | prostate | | normal | U | [Lonza CC-2555](http://www.lonza.com/) |
| ProgFib | skin | |  | M | [Progeria Research Foundation HGADFN167](http://www.progeriaresearch.org/) |
| RPTEC | epithelium | | normal | U | [Lonza CC-2553, CC-2553T225](http://www.lonza.com/) |
| RWPE1 | prostate | | normal | M | [ATCC CRL-11609](http://www.atcc.org/ATCCAdvancedCatalogSearch/ProductDetails/tabid/452/Default.aspx?ATCCNum=CRL-11609&Template=cellBiology) |
| SAEC | epithelium | | normal | U | [Lonza CC-2547](http://www.lonza.com/) |
| SK-N-MC | brain | | cancer | F | [ATCC HTB-10](http://www.atcc.org/ATCCAdvancedCatalogSearch/ProductDetails/tabid/452/Default.aspx?ATCCNum=HTB-10&Template=cellBiology) |
| SK-N-SH_RA | brain | | cancer | F | [ATCC HTB-11](http://www.atcc.org/ATCCAdvancedCatalogSearch/ProductDetails/tabid/452/Default.aspx?ATCCNum=HTB-11&Template=cellBiology) |
| SKMC | muscle | | normal | U | [Lonza CC-2561](http://www.lonza.com/) |
| SkMC | muscle | | normal | B | [PromoCell SkMC](http://www.promocell.com/nc/products/human-primary-cells/skeletal-muscle-cells/?sword_list%5B%5D=SkMC) |
| Stellate | liver | | normal | F | [Dr. Steve Choi at Duke University Stellate](http://www.dukehealth.org/physicians/steve_s_choi) |
| T-47D | breast | | cancer | F | [ATCC HTB-133](http://www.atcc.org/ATCCAdvancedCatalogSearch/ProductDetails/tabid/452/Default.aspx?ATCCNum=HTB-133&Template=cellBiology) |
| Th1 | blood | |  | U | [Dr. John Stamatoyannopoulos at UW None (primary pheresis of single normal subject)](http://www.gs.washington.edu/faculty/stamj.htm) |
| Th2 | blood | |  | U | [Dr. John Stamatoyannopoulos at UW None (primary pheresis of single normal subject)](http://www.gs.washington.edu/faculty/stamj.htm) |
| Urothelia | urothelium | | normal | F | [Dr. D Sens at University of N. Dakota Urothelia](http://pathology.med.und.nodak.edu/dept_home/MaryAnn.cfm) |
| WERI-Rb-1 | eye | | cancer | F | [ATCC HTB-169](http://www.atcc.org/ATCCAdvancedCatalogSearch/ProductDetails/tabid/452/Default.aspx?ATCCNum=HTB-169&Template=cellBiology) |
| WI-38 | embryonic lung | | normal | F | [Dr. Carl Mann, SBIGeM WI-38](http://www-dsv.cea.fr/dsv/instituts/institut-de-biologie-et-de-technologies-de-saclay-ibitec-s/unites/service-de-biologie-integrative-et-genetique-moleculaire-sbigem/laboratoire-stress-oxydants-et-cancer-lsoc/equipe-mecanismes-de-stabilite-genomique-c.-mann) |

M, male; F, female; U, not available.

**Supplementary Table 4. The list of 22 candidate genes**

| Gene | Distance from TSS | Localization in Chromosome (strain) |
| --- | --- | --- |
| DDX60L | EXON9 | chr4:169362509-169362550 (-) |
| DSE | INTRON2 | chr6:116601010-116601051 (+) |
| EDEM2 | EXON2 | chr20:33851587-33851628 (+) |
| FGD5 | EXON13 | chr3:14960296-14960337 (+) |
| MMP24 | EXON3 | chr20:33851587-33851628 (-) |
| NCOR1 | EXON2-3 | chr17:15975473-15975514 (-) |
| PODXL | EXON8 | chr7:131187824-131187865 (-) |
| UTP6 | INTRON7 | chr17:30215212-30215253 (-) |
| ZNF469 | EXON2 | chr16:88497143-88497184 (-) |
| ELF5 | EXON1 | chr11:34533332-34533373 (-) |
| ELOF1 | EXON1 | chr19:11670034-11670075 (-) |
| KBTBD6 | EXON1 | chr13:41705571-41705612 (-) |
| OTP | EXON1 | chr5:76934484-76934525 (-) |
| PLS3 | EXON1 | chrX:114795506-114795547 (+) |
| PSMB8 | EXON1 | chr6:32811778-32811819 (-) |
| SLC22A17 | EXON1 | chr14:23821451-23821492 (-) |
| SLC25A35 | EXON1 | chr17:8198153-8198194 (-) |
| SMOC2 | EXON1 | chr6:168841858-168841899 (+) |
| TCF20 | EXON1 | chr22:42609969-42610010 (-) |
| TFAP2A | EXON1 | chr6:10412536-10412577 (-) |
| TRIL | EXON1 | chr7:28997139-28997180 (-) |
| TSPYL1 | EXON1 | chr6:116601010-116601051 (-) |

**Supplementary Table 5. RNAi sequences**

| Gene | Sense sequence (5’ to 3’) |
| --- | --- |
| Negative Control | UUCUCCGAACGUGUCACGUTT |
| siZNF471-1 | CCUGGUAUCACUUGGUCUUTT |
| siZNF471-2 | GCAUGCAUGGAGGGAAUUATT |
| siPLS3-1 | CAGCAACGGAUUCAUUUGUTT |
| siPLS3-2 | CUGCUUAGAUGGGCAAACUTT |
| siTFAP2A-1 | CCAGAUCAAACUGUAAUUATT |
| siTFAP2A-2 | GGAAGAUCUUUAAGAGAAATT |
| shNC | Scrambled |
| siKAP1 | GCATGAACCCCTTGTGCTG |
| shZNF471 | CCTGGTATCACTTGGTCTTTTATGGATCCATAAAAGACCAAGTGATACCAGG |

**Supplementary Table 6. The primer list for ChIP-PCR**

| GENE | FORWARD | REVERSE |
| --- | --- | --- |
| ELF5 | TCACATGCCCAGAGTGAAGT | AATAACAGGTGTGGCTCCCA |
| ELOF1 | TGCTATTTGAGTACGCGTGC | CCAGCACTCACCTTCTCCAG |
| KBTBD6 | TGAAGATCGTGCAGCAGAAGA | GTGGCTCATCCTCTCTGGGA |
| OTP | TGCTCACGGGTTCATTCAAC | GGCATGAGACAGCATCGC |
| PLS3 | CAATCCCCTTCCTGGTTCCC | GCGCCTACAAAGCACTTACG |
| PSMB8 | GTCCAGGCACTAACTGTCCTTT | GGGGGCTCCGCATACATC |
| SLC22A17 | CCAACAGCAGGTCGGGAC | CCCAGAGCCACGAAGAGC |
| SLC25A35 | GGTTGGCTGACCGACTCATA | TGGGGCAGCTTGAAGTTGTG |
| SMOC2 | ATTTGACAGGAGCGAGGGC | CTGCAGCCGGGAGAGC |
| TCF20 | TGCCTCAACTCAGTCCAACC | AGGAAGATTGGCCACTTGAGTA |
| TFAP2A | TCGCCCACACTTTCTTTCTCA | TCTGGCGAATCACAGGGAA |
| TRIL | AACGAGATCAGCCGCCTAAG | CTTGCCCAGAAAGCGGATC |
| TSPYL1 | CGCCGCTGAAATGTTAGTGA | GATGCTGTGGGTTTGGAGG |
| GAPDH  intron | TACTAGCGGTTTTACGGGCG | TCGAACAGGAGGAGCAGAGAGCGA |

**Supplementary Table 7. Antibodies used in this study**

| Antibody | Company | Cat. No. | Dilution |
| --- | --- | --- | --- |
| PCNA | Abcam | ab29 | 1:1000 |
| Actin B | Cell Signaling Technology | 4970 | 1:2000 |
| Caspase-3 | Cell Signaling Technology | 9665 | 1:1000 |
| Caspase-7 | Cell Signaling Technology | 9492 | 1:1000 |
| Caspase-8 | Cell Signaling Technology | 9746 | 1:1000 |
| Caspase-9 | Cell Signaling Technology | 9508 | 1:1000 |
| CDK4 | Cell Signaling Technology | 12790 | 1:1000 |
| CDK6 | Cell Signaling Technology | 13331s | 1:1000 |
| Claudin-1 | Cell Signaling Technology | 4933 | 1:1000 |
| cleaved caspase 8 | Cell Signaling Technology | 9496s | 1:1000 |
| cleaved caspase 9 | Cell Signaling Technology | 9501s | 1:1000 |
| Cleaved Caspase-3 | Cell Signaling Technology | 9661 | 1:1000 |
| Cleaved Caspase-7 | Cell Signaling Technology | 9491 | 1:1000 |
| Cleaved PARP | Cell Signaling Technology | 5625 | 1:1000 |
| cyclin D1 | Cell Signaling Technology | 2922S | 1:1000 |
| E-Cadherin (4A2) | Cell Signaling Technology | 14472 | 1:1000 |
| p27 Kip1 (D69C12) | Cell Signaling Technology | 3686 | 1:1000 |
| PARP | Cell Signaling Technology | 9532 | 1:1000 |
| Slug (C19G7) | Cell Signaling Technology | 9585 | 1:1000 |
| Vimentin (D21H3) | Cell Signaling Technology | 5741 | 1:1000 |
| GAPDH | Santa Cruz | sc-25778 | 1:2000 |
| p21 (F-5) | Santa Cruz | sc-6246 | 1:1000 |
| p53 | Santa Cruz | sc-126 | 1:1000 |
| AP2 alpha | Abcam | ab52222 | 1:1000 |
| ZNF471 | Life Span | LS-C179158-100 | 1:500 |
| KAP1 | Proteintech | 15202-1-AP | 1:1000 |
| T-Plastin | Santa Cruz | sc-166208 | 1:1000 |
