# Supplementary figures and images for: Zinc-finger protein 471 suppresses gastric cancer through transcriptionally repressing downstream oncogenic PLS3 and TFAP2A
Source: Oncogene. 2018 Apr 3;37(26):3601–16. doi: 10.1038/s41388-018-0220-5 (PMC6021371; doi:10.1038/s41388-018-0220-5)

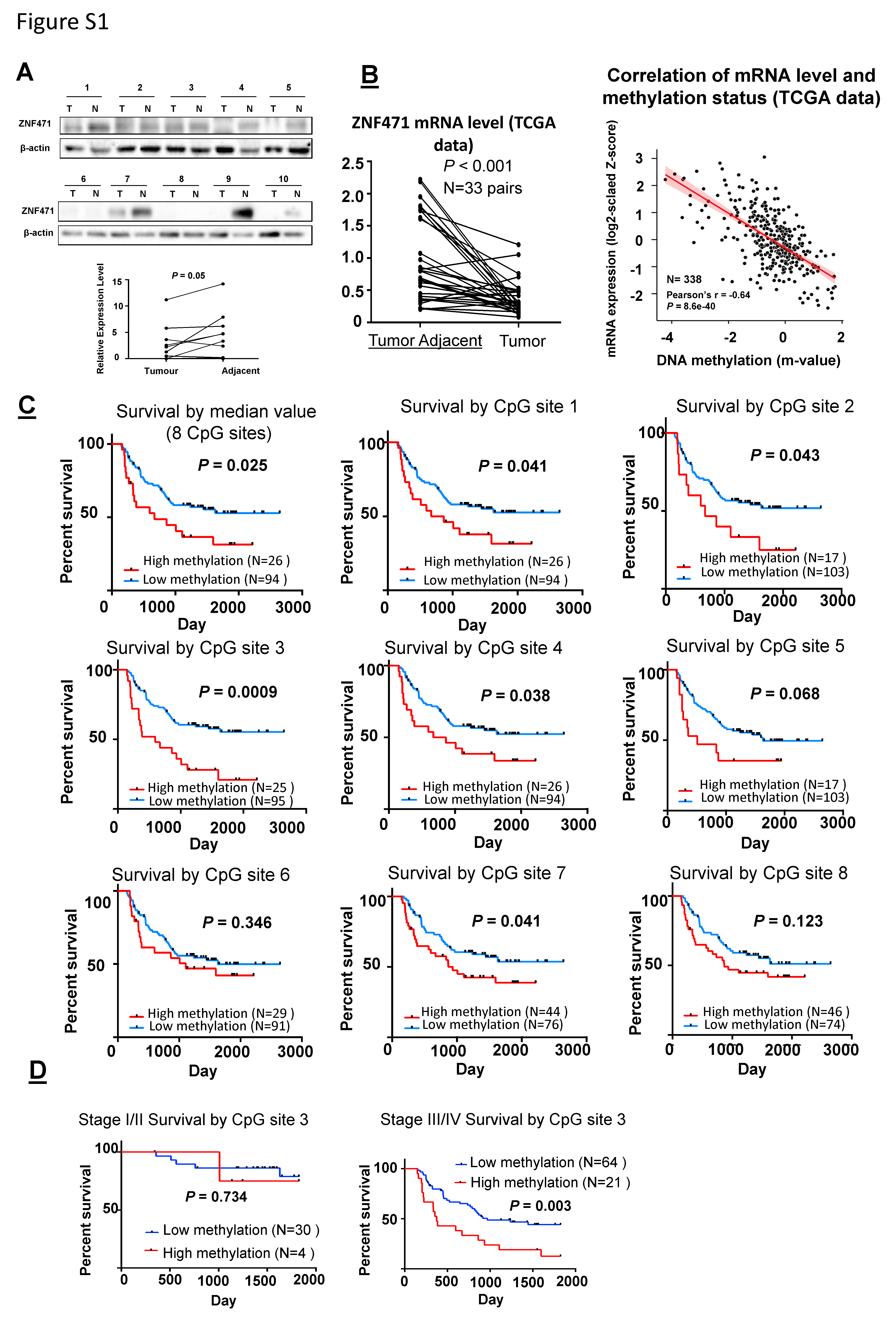

Supplement: Supplementary file 2 — Figure S1(TIF 1141 kb) [file 41388_2018_220_MOESM2_ESM.tif]

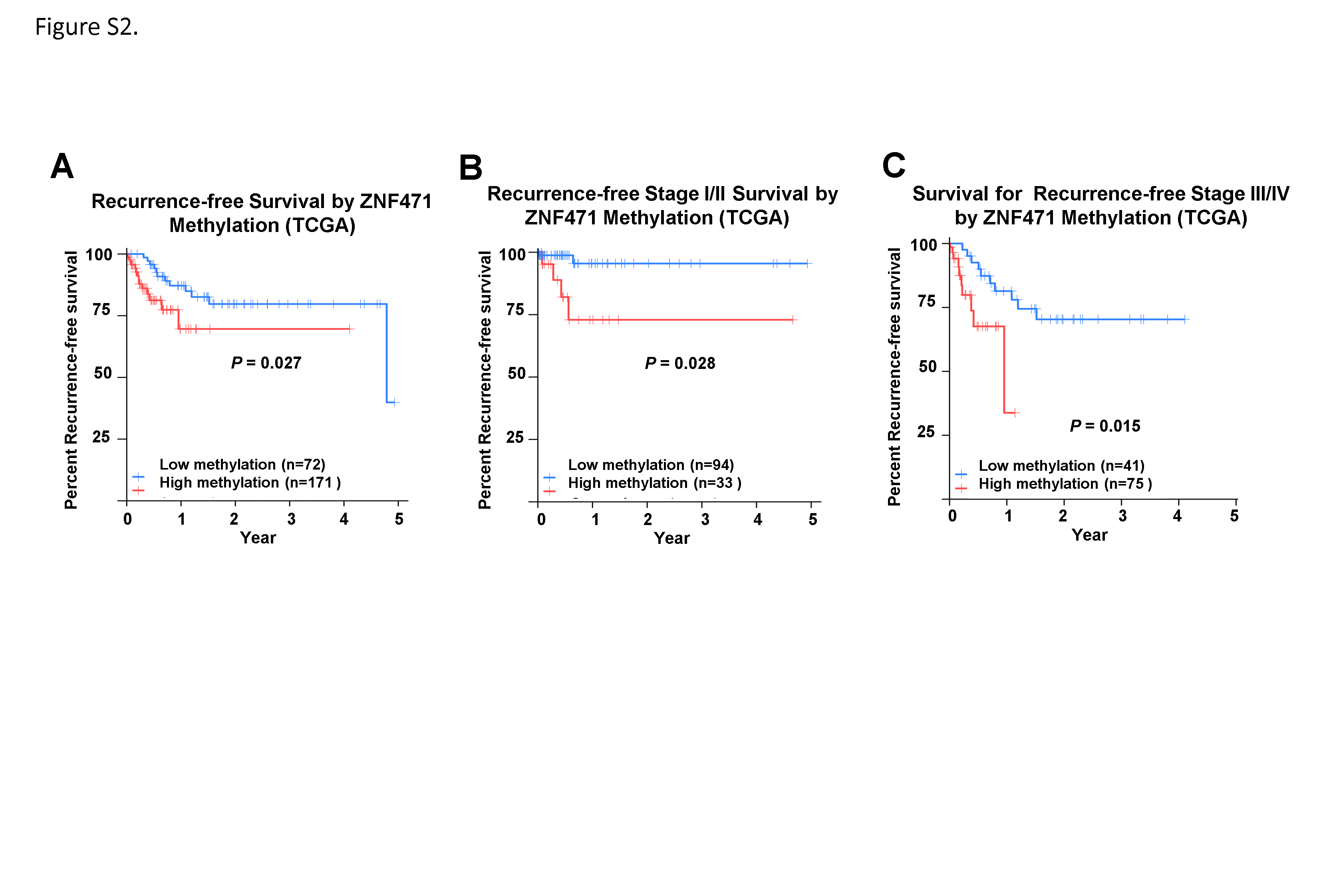

Supplement: Supplementary file 3 — Figure S2(TIF 137 kb) [file 41388_2018_220_MOESM3_ESM.tif]
