# Supplementary material for: Zinc-finger protein 471 suppresses gastric cancer through transcriptionally repressing downstream oncogenic PLS3 and TFAP2A
Source: Oncogene. 2018 Apr 3;37(26):3601–16. doi: 10.1038/s41388-018-0220-5 (PMC6021371; doi:10.1038/s41388-018-0220-5)
Supplement: Supplementary file 7 — Supplementary Information 1(DOCX 26 kb) [file 41388_2018_220_MOESM7_ESM.docx]

**Supplementary Figure legends**

**Figure S1**

**(A)** Left, the mRNA expression of ZNF471 in paired patient samples from TCGA data (n=66, *P* < 0.001). Right, the correlation between ZNF471 mRNA level and its promoter methylation status by TCGA data (n=338). **(B) Protein level of ZNF471 in paired gastric tumour and adjacent tissues by Western blot (n = 10 pairs, *P* = 0.05).** (C) Kaplan-Meier curves of patients with gastric cancer (n=120), stratified by methylation value of 8-CpG-site median and individual CpG sites of ZNF471. **(D)** Kaplan-Meier curves for gastric cancer patients (n=120) at different stages, stratified by CpG site 3 methylation status in ZNF471 promoter. Left, patients in stage I/II (n=34); Right, patients in stage III/IV (n=86).

**Figure S2**

(A) Kaplan-Meier curves for recurrence-free survival of gastric cancer patients (n=243) in TCGA database. (B) Kaplan-Meier curves for recurrence-free survival of gastric cancer patients (n=127) from Stage I/II in TCGA database. (C) Kaplan-Meier curves for recurrence-free survival of gastric cancer patients (n=116) from Stage III/IV in TCGA database.

**Figure S3**

**(A)** Cell doubling time for ZNF471 overexpression cell lines, AGS, BGC823 and MKN74. **(B)** Cell doubling time for GES1 and MKN1 with ZNF471 knockdown by siRNA. Data are expressed as mean ± S.D.

**Figure S4**

**(A)** EMSA performed with the biotin-labeled PSMB8 probes and 293T nuclear extracts with overexpressed ZNF47-Falg. (**B)**Top 10 pathways enriched in PSMB8 high-expression samples from TCGA gastric cancer database by GSEA. **(C)** The siRNA knockdown efficiency for TFAP2A in AGS, BGC823 and MKN74 by Western blot. **(D)** The siRNA knockdown efficiency for PLS3 in BGC823 and MKN74 by Western blot. **(E)** The effect of PLS3 knockdown on cell viability in BGC823 and MKN74 by siRNA. **(F)** The siRNA knockdown efficiency for KAP1 in AGS, MKN74 and GES1 by Western blot. Data are expressed as mean ± S.D. ns, not significant.

**Figure S5. The binding affinity between ZNF471 and downstream gene TFAP2A and PLS3. (A)** The plot and the EMSA result show the fraction of TFAP2A probe bound as nuclear extracts was titrated. K_D_ = 388 ± 37.3 ng/µl, B_max_ = 1.214. **(B)** The plot and the EMSA result show the fraction of PLS3 probe bound as nuclear extracts was titrated. K_D_ = 392 ± 28.78 ng/µl, B_max_ = 0.959.

**Supplementary Methods**

**Cell doubling time**

To measure the doubling time of cells with ZNF471, shZNF471/siZNF471, we seeded the transfected cells to the 12-well plate at the density of 2 × 10^4^ cells/ml. The number of cells was counted every 24h for 4 days and the doubling time was then calculated with the following formula:

$Doubling Time =\frac{Duration\times log(2)}{\log\left( Final Concentration \right)-log(Initial Concentration)}$ ,

where "log" is the logarithm to base 10 or 2 or any other base.

**Equilibrium & dissociation constants determination**

Binding affinity for a transcription factor and its target DNA is used to describe the strength of the binding interaction, and typically represented by equilibrium dissociation constant (*K*_D_), the concentration of transcription factor protein bounded with 50% DNA as a complex. To measure the *K*_D_ for ZNF471 and TFAP2A/PLS3, EMSA was adopted to monitor the binding. Nuclear extracts were prepared from 293T cells transfected with pCDNA3.1-ZNF471 for 48h with NE-PER Nuclear and Cytoplasmic extraction kit (Thermo Fisher Scientific). The biotin labeled 42-bp dsDNA was prepared by annealing two complementary HPLC-purified DNA oligosin annealing buffer (10 mM Tris, 1 mM EDTA, 50 mM NaCl, pH 8.0) at a concentration of 1 pmol/µl in a temperature gradient of 0.1°C/s from 95°C to 26°C.

The EMSA reaction was assembled on ice a with 25 fmol DNA probe for each reaction and various concentration of nuclear extracts in binding buffer (10mM Tris, 50mM KCl, 1mM DTT, 50ng/μl Poly (dΙ•dC); pH 7.5). Following the EMSA, the fraction of DNA bound was determined from the background-subtracted signal intensities using the expression: bound/(bound + unbound). Then the fraction of DNA bound in each reaction was plotted versus the concentration of nuclear extracts. The data were fit with the following binding equation using Prism software to perform non-linear regression and obtain a value for *K*_D_ and B_max_.
