# Supplementary material for: Zinc-finger protein 471 suppresses gastric cancer through transcriptionally repressing downstream oncogenic PLS3 and TFAP2A
Source: Oncogene. 2018 Apr 3;37(26):3601–16. doi: 10.1038/s41388-018-0220-5 (PMC6021371; doi:10.1038/s41388-018-0220-5)

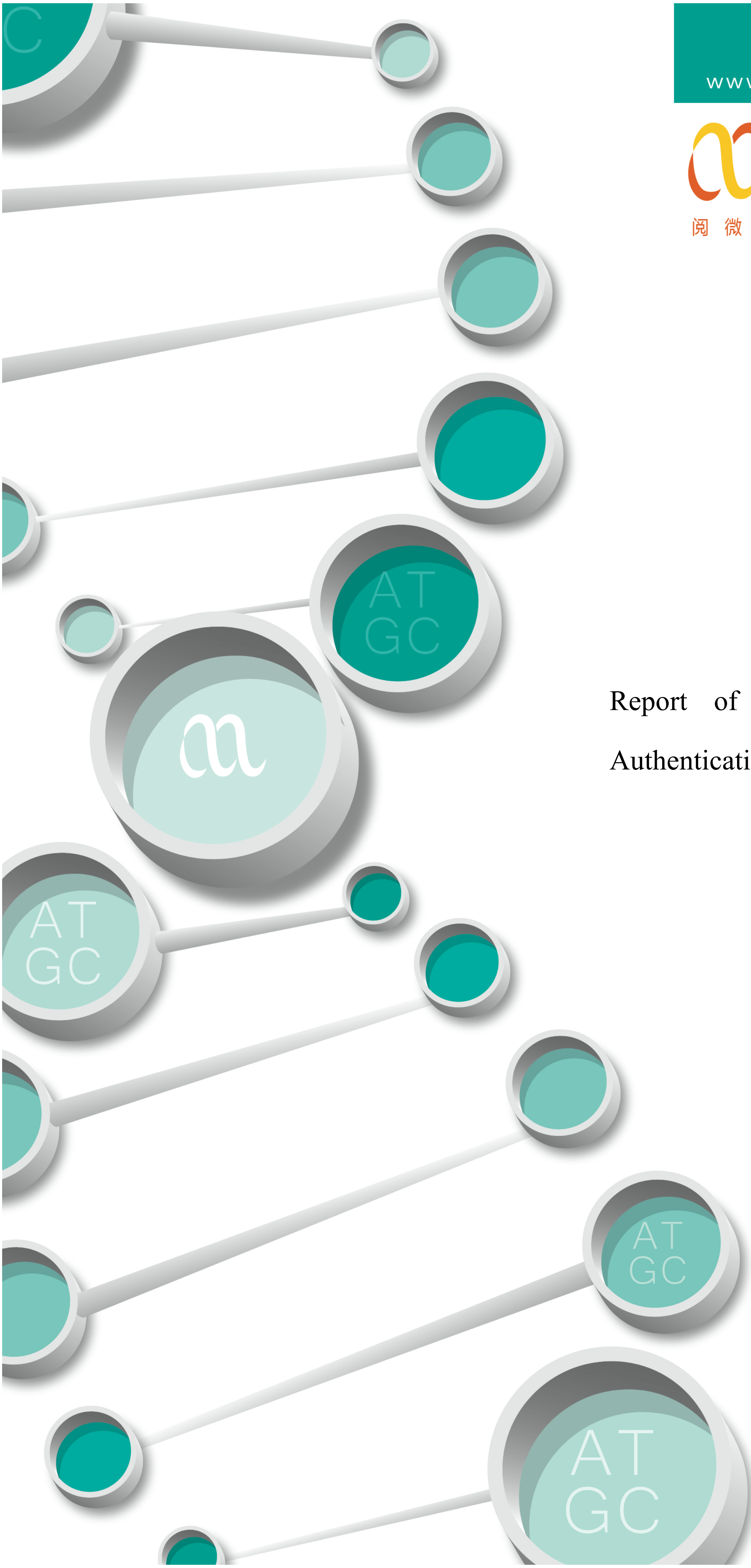

www.microread.com

阅微基因
icroread
阅 微 基 因 省 时 省 心

Report of Human Cell Line Authentication

Report of Human Cell Line Authentication

Delivery Date: November 02nd, 2017

Analysis Date: November 09th, 2017

Ⅰ.Sample

Sample Name: ‘XB6528’, labeled as ‘MKN28’, was received on November 02nd, 2017.

Ⅱ.Methodand Procedure

1. PCR is amplified with STR Multi-amplification Kit (Microreader™21 ID System);
2. PCR products are assayed with ABI 3730xl DNA Analyzer (Applied Biosystems®).
3. Data were analyzed using GeneMapper3.2 software and then compared with the ATCC and DSMZ databases for reference matching.

Ⅲ. Results

1. The results of the negative and positive control match expectations.
2. The STR profiles of the cell line sample are in the attached table and figure.

Genetic Site	ATCC				Customer sample				
(Locus)	Database profile：Het-1AEsophageal EpitheliumHuman				Query profile：MKN28				
Amelogenin	X				X				
D5S818	11	12			11				
D13S317	11				11				
D7S820	9				9				
D16S539	9	11			9	11			
vWA	16				16	20			
TH01	7				6				
TPOX	11				8	11			

CSF1PO	10	12			12				
Number of shared alleles between query sample and database profile									9
Total number of alleles in the database profile									13
Percent match between the query and the database profile：									69%

Sample “MKN28”: (1) No cross-contamination of other human cell line is found.
(2) The submitted profile is an 69% match for the following ATCC human cell line(s) in the ATCC STR database (8 core loci plus Amelogenin): Het-1AEsophageal EpitheliumHuman.
(3) 100% matched cell line is MKN74 found in JCRB Cell Bank.

Operator: Rui Wang　　　　　　　　Auditor: Bingjun Wu

Beijing Microread Genetics Co., Ltd

Notes：

1. Based on the ANSI Standard, cell lines with ≥80% match are considered to be related; i.e.,derived from a common ancestry. Cell lines with between a 55% to 80% match require further profiling for authentication of relatedness.

2. The short tandem repeat (STR) profile generated by Beijing Microread Genetics Co., Ltd is indicative only of the sample sent to Beijing Microread Genetics Co., Ltd at the time it was sent. This data and analysis are for research use only.

Table: STR profiles of MKN28 cell line

Cell line MKN28（Fig.XB6528）		
Marker	Allele 1	Allele 2
D19S433	13	15.2
D5S818	11	11
D21S11	32.2	33.2
D18S51	12	12
D6S1043	13	13
AMEL	X	X
D3S1358	16	16
D13S317	11	11
D7S820	9	9
D16S539	9	11
CSF1PO	12	12
Penta D	9	9
D2S441	10	12
vWA	16	20
D8S1179	11	16
TPOX	8	11
Penta E	11	14
TH01	6	6
D12S391	18	21
D2S1338	18	23
FGA	23	23

Figure: STR profiles of MKN28 cell line

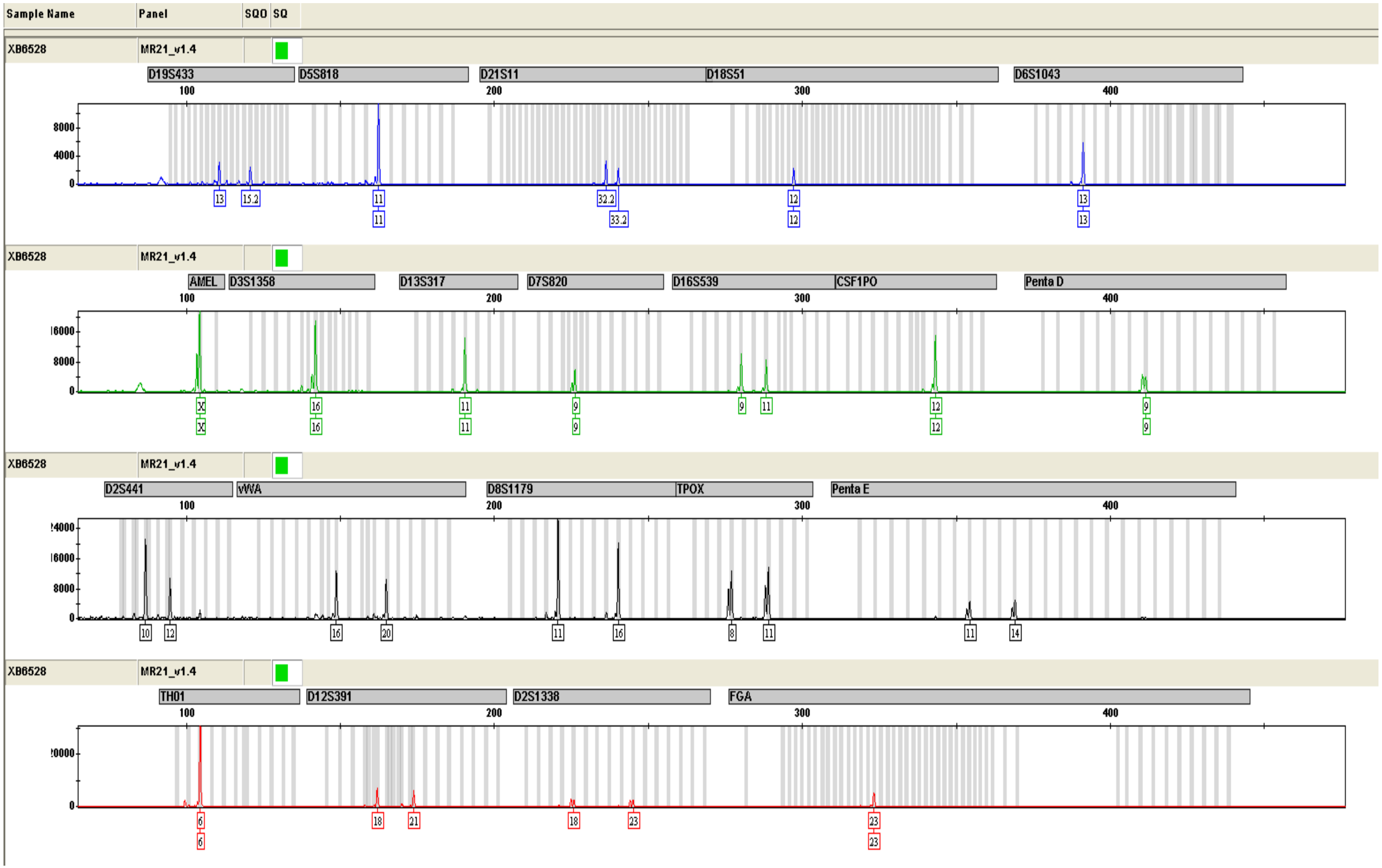

Supplement: Supplementary file 8 — Supplementary Information 2(PDF 2156 kb) [file 41388_2018_220_MOESM8_ESM.pdf]
